# Clinical outcome assessment trends in clinical trials—Contrasting oncology and non‐oncology trials

**DOI:** 10.1002/cam4.6325

**Published:** 2023-07-08

**Authors:** Yeonju Kim, Mark R. Gilbert, Terri S. Armstrong, Orieta Celiku

**Affiliations:** ^1^ Neuro‐Oncology Branch National Cancer Institute, National Institutes of Health Bethesda Maryland USA

**Keywords:** clinical trials, oncology, patient outcome assessment, patient‐reported outcomes, quality of life

## Abstract

**Background:**

Clinical outcome assessments (COAs) are key to patient‐centered evaluation of novel interventions and supportive care. COAs are particularly informative in oncology where a focus on how patients feel and function is paramount, but their incorporation in trial outcomes have lagged that of traditional survival and tumor responses. To understand the trends of COA use in oncology and the impact of landmark efforts to promote COA use, we computationally surveyed oncology clinical trials in ClinicalTrials.gov comparing them to the rest of the clinical research landscape.

**Methods:**

Oncology trials were identified using medical subject heading neoplasm terms. Trials were searched for COA instrument names obtained from PROQOLID. Regression analyses assessed chronological and design‐related trends.

**Results:**

Eighteen percent of oncology interventional trials initiated 1985–2020 (*N* = 35,415) reported using one or more of 655 COA instruments. Eighty‐four percent of the COA‐using trials utilized patient‐reported outcomes, with other COA categories used in 4–27% of these trials. Likelihood of COA use increased with progressing trial phase (OR = 1.30, *p* < 0.001), randomization (OR = 2.32, *p* < 0.001), use of data monitoring committees (OR = 1.26, *p* < 0.001), study of non‐FDA‐regulated interventions (OR = 1.23, *p* = 0.001), and in supportive care versus treatment‐focused trials (OR = 2.94, *p* < 0.001). Twenty‐six percent of non‐oncology trials initiated 1985–2020 (*N* = 244,440) reported COA use; they had similar COA‐use predictive factors as oncology trials. COA use increased linearly over time (*R* = 0.98, *p* < 0.001), with significant increases following several individual regulatory events.

**Conclusion:**

While COA use across clinical research has increased over time, there remains a need to further promote COA use particularly in early phase and treatment‐focused oncology trials.

## Graphical abstract

Comprehensive evaluation of interventional clinical trials in the ClinicalTrials.gov registry revealed low but increasing rates of clinical outcome assessment incorporation in oncology trials. While the study revealed a positive impact of regulatory and advocacy efforts on increasing use of clinical outcome assessments, there remains a need to further target early phase and treatment‐focused oncology trials.

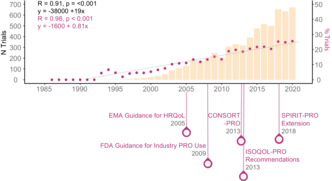

## 1 INTRODUCTION

Clinical outcome assessments (COAs) are measures of how patients feel, function, and survive.^1^ COAs play a crucial role in holistic evaluation of patient care and can be particularly informative in oncology where the multitude of symptoms, treatment‐related toxicities, and often poor prognosis of patients necessitate a focus on symptom management and quality of life. Landmark clinical trials have demonstrated that incorporation of COAs in trial outcomes can inform whether the intervention benefit is meaningful,^2^ that COAs can capture within‐patient effects,^3^ and that patient reporting of symptoms can even lead to prolonged survival.^4^ Therefore, designing oncology trials to incorporate COAs in conjunction with survival, tumor response, and safety outcomes is key to patient‐centered assessment of net clinical benefit of novel interventions and supportive care.

COAs include four categories: patient‐reported outcomes (PRO), observer‐reported outcomes (ObsRO), clinician‐reported outcomes (ClinRO), and performance outcomes (PerfO) measures, which serve complementary roles in clinical trials.^1^ PROs and PerfOs enable multifaceted assessments of treatment toxicity and efficacy whereas ClinROs serve in determining trial eligibility and treatment course, and as correlates to objective outcomes and disease progression.^5^ COAs presently encompass over 2500 individual instruments and 300 composite measures which range in scope and applicability, with indications covering from specific conditions and neoplasms to all areas of clinical care.^6^ This diversity has invariably presented its own challenges, including the difficulty in selecting valid, reliable, and clinically useful instruments, since quality of life and other constructs of interest are often multidimensional, dynamic, and unique to an individual or situation.^5^ Regulatory agencies have recognized the value and challenges of incorporating COAs in drug development,^7^, ^8^ and multiple initiatives and guidelines for trial design, analysis, and reporting advocate for the use of COAs across clinical research.^9^, ^10^, ^11^, ^12^

PRO use has been evaluated in general oncology trials and focused cancer trial subsets^13^, ^14^, ^15^, ^16^, ^17^, ^18^ but the comprehensive use of COAs in oncology trials across time and how it compares to the use of COAs in non‐oncology clinical trials has not been evaluated. Moreover, trends in relation to landmark regulatory and advocacy events have not been assessed, and with few exceptions^19^, ^20^ previous evaluations relied on manual curation of the literature which is not feasible for periodic assessments. In this study, we extensively evaluated COA use across oncology trials and how it compares to the COA use in the rest of clinical trials in the ClinicalTrials.gov registry. We leveraged the registry data through programmatic semi‐automated means and assessed the impact of key regulatory and advocacy events.

## 2 METHODS

### 2.1 Selecting and obtaining study data

ClinicalTrials.gov registry was accessed programmatically on August 1, 2021 using R (*RPostgreSQL* library) through the Aggregate Analysis of ClinicalTrials.gov database.^21^, ^22^ Interventional trials with start date 1985–2020 were retrieved from the registry. Oncology trials were determined by searching for Medical Subject Headings^23^ terms under “Neoplasms by Site” (Table S1) in study title, description, or design. The European Union (EU) or The United States (US)‐based trials were determined by site country location (Table S2). Trial data obtained included initiation year, phase, primary purpose, intervention model, randomization, blinding, data monitoring committee (DMC) use, regulatory status of drug or device, and primary sponsors.

### 2.2 Obtaining clinical outcome assessment information

COA instrument details including name, acronyms, COA type (PRO, ClinRO, PerfO, ObsRO, or composite), and therapeutic indications, were extracted from PROQOLID^6^ using Python's *BeautifulSoup* library on October 26, 2020. Several COAs with ambiguous acronyms (such as QLQ or QOL) or ambiguous meanings in trials (e.g., PFS referring either to the Parkinson Fatigue Scale or Progression Free Survival) were excluded from search terms to improve specificity. COA use in trials was identified by searching for COA instrument names and acronyms among trial entries relevant to outcome measures. COA use was further categorized into primary, secondary, or other outcome by matching COA instrument names with trial outcome design descriptions. Similarly, trials assessing overall survival, progression free survival, and tumor response endpoints were identified by searching for relevant terms among trial entries relevant to outcome measures.

### 2.3 Trends of COA use over time

Trends in COA use over time were assessed using Pearson's correlation and linear regression analysis between trial initiation year and the proportion of trials using COAs. When stratified by trial primary purpose or COA category, the proportion was calculated with respect to the baseline count of all (oncology or non‐oncology) trials and all (oncology or non‐oncology) trials using COAs, respectively. Years with statistically outlying (beyond three times the interquartile range) proportion of trials using COAs were excluded from regression. We removed outlier years with regression standardized residuals larger than three and representing years with fewer than 1% of trials to avoid skew in COA use rates from years with low trial counts and confirmed that regression residuals were normally distributed (Shapiro–Wilk deviation from normality: *W* = 0.96, *p* = 0.204) and not dependent on year.

### 2.4 COA use and landmark events

Chi‐square tests with continuity correction were used to compare COA use in trials initiated before versus after five landmark events driven by regulatory bodies and initiatives including the European Medicines Agency (EMA), Food and Drug Administration (FDA), Consolidated Standards of Reporting Trials (CONSORT), International Society for Quality of Life Research (ISOQOL), and Standard Protocol Items: Recommendations for Interventional Trials (SPIRIT). Logistic regression was used to assess linear trends between COA use with binary indicator variables for events (whether the trial took place before or after the event) and trial initiation year as a continuous variable and an interaction term between the two covariates. False discovery rate multiple hypothesis correction was performed across models and covariates.

### 2.5 Trial characteristics associated with COA use

Logistic regression was performed to investigate associations between COA use and trial characteristics: phase (as ordinal variable), intervention FDA regulatory status, lead sponsor and collaborator class (National Institutes of Health (NIH) or other US federal agencies, industry, or other, which includes trial networks, academic institutions, and individual investigators), primary purpose (treatment, supportive care, diagnostic, or other, which includes basic science, health services research, prevention, screening, device feasibility, educational/counseling/training), blinding, number of trial arms (single‐ or multi‐arm), data monitoring committee (DMC) use, and randomization.

Analyses were conducted using R version 3.6.0.^24^

## 3 RESULTS

### 3.1 Trial characteristics and associations with COA use

**FIGURE 1:**
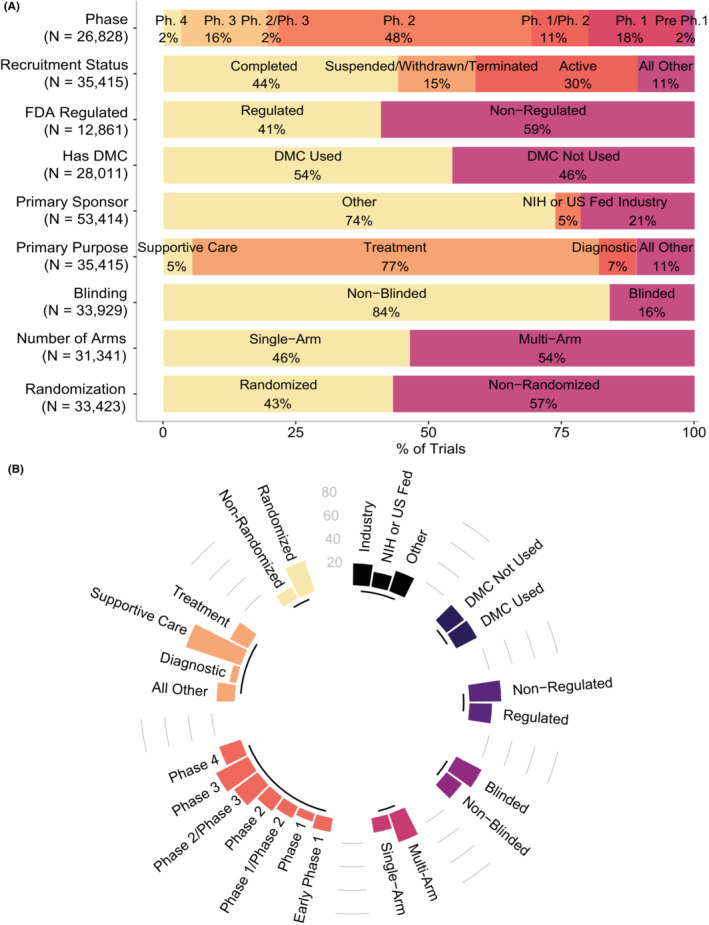
Characteristics of interventional oncology trials and associations with clinical outcomes assessment (COA) use. (A) Baseline characteristics of interventional oncology trials. (B) Proportion of COA use by trial characteristics. *N* indicates the number of trials using a COA in each category.

A total of 279,855 interventional trials with start dates between 1985 and 2020 (a date range chosen to encompass 95% of all interventional trials in the registry) were retrieved from the registry: 35,415 were determined to be oncology trials studying one or more neoplasms, and the remaining 244,440 were considered non‐oncology trials. The design features and the number of trials reporting them are summarized in Figure 1A and Table S3. Briefly, the oncology trials reporting relevant information most commonly had a primary purpose of treatment (77%), studied a non‐FDA‐regulated intervention (59%), were completed (44%), phase 2 (48%), non‐blinded (84%), multi‐arm (54%), and non‐randomized (57%). Similarly, non‐oncology trials predominantly had a primary purpose of treatment (64%) and studied a non‐FDA‐regulated intervention (72%), but they had more balanced phase distribution (with phase 1, phase 2, and phase 3 at 22%, 25%, and 21%, respectively), and a higher degree of blinding (52% non‐blinded), multi‐arm design (77%), and randomization (70%) (Figure S1; Table S3).

Eighteen percent of oncology trials reported COA use as determined by the search for individual instruments among trial design entries related to outcomes. Early phase trials had relatively lower rates of COA use (less than 15% each) compared to phase 2/phase 3 (25%), phase 3 (30%), and phase 4 (19%) trials. Nineteen percent of trials studying an FDA‐regulated intervention used a COA, versus 27% among trials studying non‐FDA‐regulated interventions. Eighteen percent of industry‐sponsored trials used COAs, versus 13% of NIH and US federal government‐sponsored, and 18% of all other sponsors. Trials focused on supportive care had the greatest rate of COA use (49%), followed by trials focused on treatment (17%), diagnostic (6%), with cumulative other trial categories having 15% COA use. Trials with blinding had greater rates of COA use (29%) than open‐label trials (16%). Multi‐arm trials had greater rates of COA use (25%) compared to single‐arm trials (12%). Twenty‐seven percent of randomized trials used COAs compared to 12% in non‐randomized trials (Table 1).

**TABLE 1:** Rate of clinical outcomes assessment (COA) use and associations with trial characteristics via logistic regression.

Trial characteristics	COA use			
	Oncology (*N* = 7339 reporting all data)		Non‐oncology (*N* = 31,481 reporting all data)	
	% using COAs (*n*)	OR (*p*)	% using COAs (*n*)	OR (*p*)
Phase (ordinal)	*N* = 26,828^a^	**1.30 (<0.001)**	*N* = 132,379	**1.17 (<0.001)**
Early phase 1	11.71% (65/555)	–	19.38% (534/2756)	–
Phase 1	6.89% (331/4806)	–	8.71% (2504/28,749)	–
Phase 1/phase 2	10.87% (314/2889)	–	20.93% (1749/8355)	–
Phase 2	14.81% (1888/12,745)	–	26.02% (8708/33,461)	–
Phase 2/phase 3	24.83% (144/580)	–	28.11% (1390/4944)	–
Phase 3	30.4% (1325/4358)	–	30.22% (8373/27,711)	–
Phase 4	19.22% (172/895)	–	23.98% (6331/26,403)	–
FDA regulated drug/device	*N* = 12,861		*N* = 91,120	
Non‐regulated	26.97% (2048/7593)	–	32.65% (21,452/65,705)	–
Regulated	19.08% (1005/5268)	**0.81 (0.001)**	28.02% (7121/25,415)	1.03 (0.271)
Has DMC	*N* = 28,011		*N* = 203,047	
DMC not used	18.54% (2368/12,774)	–	25.8% (31,924/123,719)	–
DMC used	19.78% (3014/15,237)	**1.26 (<0.001)**	28.42% (22,544/79,328)	**1.26 (<0.001)**
Primary sponsor	*N* = 35,414		*N* = 244,345	
Industry	18.41% (1400/7606)	–	22.85% (15,989/69,988)	–
NIH or US fed	12.71% (213/1676)	1.16 (0.377)	19.9% (1714/8613)	**1.39 (<0.001)**
Other	18.05% (4718/26,132)	0.89 (0.089)	27.05% (44,841/165,744)	**0.89 (<0.001)**
Primary purpose	*N* = 35,415		*N* = 244,440	
Supportive care	49.22% (946/1922)	–	36.95% (3854/10,429)	–
Treatment	17.13% (4648/27,128)	**0.34 (<0.001)**	29.53% (46,126/156,225)	1.16 (0.100)
Diagnostic	5.78% (144/2493)	**0.12 (<0.001)**	11.27% (1117/9915)	**0.47 (<0.001)**
All other	15.32% (593/3872)	**0.26 (<0.001)**	16.87% (11,447/67,871)	**0.46 (<0.001)**
Blinding	*N* = 33,929		*N* = 240,886	
Blinded	29.21% (1583/5419)	–	30.83% (35,439/114,956)	–
Non‐blinded	16.32% (4653/28,510)	0.85 (0.059)	21.33% (26,861/125,930)	**0.55 (<0.001)**
Number of arms	*N* = 31,341		*N* = 224,791	
Multi‐arm	25.27% (4245/16,799)	–	28.23% (49,053/173,763)	
Single‐arm	12.32% (1792/14,542)	1.22 (0.056)	21.35% (10,895/51,028)	**1.26 (<0.001)**
Randomization	*N* = 33,423		*N* = 238,652	
Non‐randomized	12.16% (2308/18,975)	–	20.45% (14,542/71,124)	
Randomized	27.26% (3938/14,448)	**2.32 (<0.001)**	28.38% (47,537/167,528)	**1.3 (<0.001)**
Intercept		0.21 (<0.001)		0.19 (<0.001)

*Note*: Bolded CI indicate statistical significance at α = 0.05.

Abbreviations: DMC, data monitoring committee; OR, odds ratio; US Fed, US Federal Government; –, phase was defined as an ordinal variable for the regression and for all other characteristics, indicates reference value in logistic regression.

^a^ Number of trials reporting trial characteristic.

We next evaluated the interplay of the trial design elements in the implementation of COAs using multi‐variate logistic regression across trials reporting sufficient information (*N* = 7339; Table 1). The likelihood of COA use increased significantly with progressing clinical trial phases (OR = 1.30, *p* < 0.001). Trials studying FDA‐regulated interventions were significantly less likely to use COAs than those studying non‐FDA‐regulated interventions (OR = 0.81, *p* = 0.001) whereas trials using DMCs were more likely to use COAs than those without DMCs (OR = 1.26, *p* < 0.001). Compared to the baseline rate of COA use among supportive care trials, trials focused on treatment (OR = 0.34, *p* < 0.001), diagnostic (OR = 0.12, *p* < 0.001), and all other purposes (OR = 0.26, *p* < 0.001) were significantly less likely to use COAs. Randomized trials were significantly more likely to use COAs than non‐randomized studies (OR = 2.32, *p* < 0.001; Figure 1B). Primary sponsor class, blinding, and single‐ versus multi‐arm design were not significantly associated with COA use.

Non‐oncology trials in the registry had higher rates of COA use overall (26%) and similar trends in COA use by phase, DMC use, and randomization, whereas unlike in oncology trials, treatment‐focused trials had similar rates of COA use compared to supportive care studies (OR = 1.16, *p* = 0.100). Additionally, compared to industry‐sponsored trials, trials with other non‐classified sponsors were less likely to use COAs than industry‐sponsored trials (OR = 0.89, *p* < 0.001), while trials with sponsorship from NIH or US federal government (versus industry; OR = 1.39, *p* < 0.001), blinding schemes (OR = 1.82, *p* < 0.001), and single‐arm designs (OR = 1.26, *p* < 0.001) were significantly more likely to use COAs. The FDA regulatory status of the trial intervention was not significantly associated with COA use among non‐oncology trials (Table 1).

### 3.2 Characteristics of COA instruments used

We identified 655 unique instruments used across the oncology trials, representing a diverse set of COAs and comprising of 24% of all instruments obtained from PROQOLID (*N* = 2695). The top instruments in order of frequency were the European Organization for Research and Treatment of Cancer Quality of Life Questionnaire‐Core Questionnaire (EORTC QLQ‐C30) used by 29%, Eastern Cooperative Oncology Group (ECOG) Performance Status used by 14%, Euroqol EQ‐5D used by 10%, Hospital Anxiety and Depression Scale (HADS) used by 5%, and Brief Pain Inventory (BPI) used by 4% of trials using COAs (Table S4). Fifty‐six percent of trials using COAs reported using one instrument (range: 1–13 instruments per trial). Of trials reporting details of trial outcomes (*N* = 5795), COAs were used predominantly as a secondary trial outcome (84%), followed by primary trial outcome (23%), and other outcome (7%). Non‐oncology trials reported a broader range of instruments encompassing 1709 unique COAs; most frequently used instruments were the SF‐36 Health Survey (8%), EQ‐5D (6%), Patient Health Questionnaire (4%), and HADS (3%) (Table S5). Similarly to oncology, 50% of non‐oncology trials using COAs reported using one instrument (range: 1–19). Compared to oncology, a greater proportion of non‐oncology trials reported COAs as a primary outcome (49%), with 75% and 7% of trials reporting COAs as secondary and other outcome.

**FIGURE 2:**
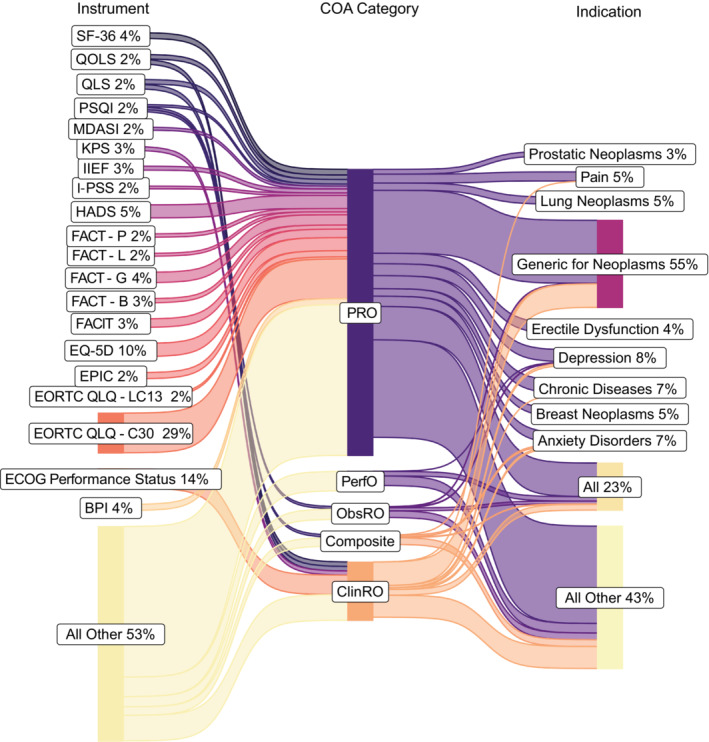
Top clinical outcomes assessments used by trials and their indication.

**FIGURE 3:**
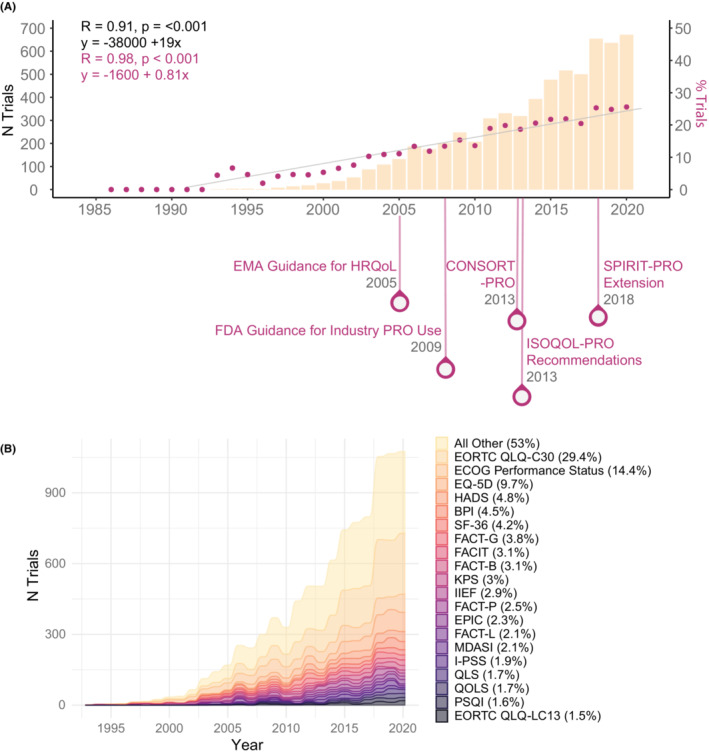
Trends of clinical outcomes assessment (COA) use over time among interventional oncology trials. (A) Linear trend in the number and proportion of trials using COAs across the timeline of notable events for COAs in clinical trials. (B) Trends in use of top COAs over time.

Among the COA categories, PROs were used in 84% of oncology trials reporting COA use (*N* = 6331), followed by ClinROs in 27%, ObsROs in 6%, PerfOs in 4%, while 4% used composite measures. Oncology trials most often utilized COAs indicated as generic for neoplasms (63%), all conditions (32%), chronic diseases (10%), depression (8%), anxiety disorders (8%), pain (7%), breast neoplasms (6%), and lung neoplasms (5%); all other COA indication groups were represented in less than 5% of trials using COAs (Figures 2 and 3B). Among non‐oncology trials reporting COA use (*N* = 62,544), 78% used PROs, 43% used ClinROs, 13% used ObsROs, 11% used PerfOs, and 16% used composite measures. These instruments were indicated for all conditions (47%), depression (16%), anxiety disorders (13%), schizophrenia (9%), generic for mental disorders (9%), generic for neoplasms (6%), Parkinson's disease (6%), pain (6%), and generic for psychiatry/psychology (5%), with all other indications representing less than 5% of trials using COAs (Figures S2 and S3B).

Stratifying oncology trials by primary purpose, treatment‐focused trials (*N* = 4648 reporting COAs) most commonly used oncology‐specific quality of life measures (EORTC QLQ‐C30 in 33%), measures of overall health status (ECOG Performance Status in 18%), and other quality of life measures (EQ‐5D in 11%); all other instruments were reported in fewer than 5% of trials (Table S4). Among trials with a primary purpose of supportive care (*N* = 946 reporting COAs), the EORTC QLQ‐C30 (20%) and EQ‐5D (6%) were also among the most commonly used; additionally, a broader range of COAs were more prevalent, including HADS (16%), Functional Assessment of Chronic Illness Therapy (8%), Functional Assessment of Cancer Therapy‐General (8%), Short Form Health Survey (8%), BPI (7%), Functional Assessment of Cancer Therapy‐Breast Cancer (6%), Center for Epidemiologic Studies Depression Scale (5%), and Pittsburgh Sleep Quality Index (5%) (see Tables S4 and S5 for top COAs used for trials of other purpose and non‐oncology trials).

### 3.3 Trends of COA use over time

We assessed the landscape of COA use over time using linear regression, revealing that both the number and proportion of oncology trials using COAs increased significantly over time (*R* = 0.91, *p* < 0.001 and *R* = 0.98, *p* < 0.001, respectively), at rates of an additional 19 trials per year (95% Confidence Interval (CI): 16–22) and 0.81% of trials per year (95% CI: 0.75%–0.88%/year), respectively (Figure 3A). The increase was observed for all trial subsets stratified by primary purpose (treatment: +0.80% trials/year, 95% CI: 0.74–0.87%/year; diagnostic: +0.28% trials/year, 95% CI: 0.18–0.37%/year, supportive care: +2.40% trials/year, 95% CI: 1.95–2.84%/year; and cumulative all other: +0.75% trials/year, 95% CI: 0.61–0.89%/year). Moreover, the use of each of the five COA categories increased significantly over time. PROs had the greatest rate of increase with an additional 0.72% of trials using PROs per year (95% CI: 0.64–0.81%/year), followed by ClinROs (+0.20% trials/year, 95% CI: 0.18–0.22%/year), ObsROs (+0.05% trials/year, 95% CI: 0.04–0.06%/year), PerfOs (+0.04% trials/year, 95% CI: 0.03–0.05%/year), and composite measures (+0.04% trials/year, 95% CI: 0.03–0.05%/year) (Table 2 and Figure S4A).

The proportion of non‐oncology trials using COAs also increased significantly over time (*R* = 0.98, *p* < 0.001), at a rate of 0.94% of trials per year (95% CI: 0.87%–1.02%/year). Stratification by trial purpose and COA type revealed similar significant increases in COA use compared to baseline across all levels (Figures S3A and S4B; Table S6).

**TABLE 2:** Correlation analysis and linear regression between number and proportion of trials using clinical outcomes assessments (COAs) each year, stratified by COA type and primary purpose of trials.

	*N* trials over time		% of trials over time	
	*R* (*p*)	*β* (trials per year; 95% CI)	*R* (*p*)	*β* (% of trials per year; 95% CI)
COA type				
PRO	**0.89 (<0.001)**	**16.80 (13.83, 19.78)**	**0.95 (<0.001)**	**0.72 (0.64, 0.81)**
ClinRO	**0.94 (<0.001)**	**4.68 (4.10, 5.26)**	**0.95 (<0.001)**	**0.20 (0.18, 0.22)**
PerfO	**0.81 (<0.001)**	**0.89 (0.66, 1.11)**	**0.86 (<0.001)**	**0.04 (0.03, 0.05)**
ObsRO	**0.87 (<0.001)**	**1.17 (0.93, 1.40)**	**0.91 (<0.001)**	**0.05 (0.04, 0.06)**
Composite	**0.82 (<0.001)**	**0.87 (0.65, 1.09)**	**0.88 (<0.001)**	**0.04 (0.03, 0.05)**
Primary purpose				
Treatment	**0.92 (<0.001)**	**13.80 (11.74, 15.87)**	**0.97 (<0.001)**	**0.80 (0.74, 0.87)**
Supportive care	**0.92 (<0.001)**	**4.22 (3.51, 4.94)**	**0.92 (<0.001)**	**2.40 (1.95, 2.84)**
Diagnostic	**0.88 (<0.001)**	**0.52 (0.41, 0.63)**	**0.74 (<0.001)**	**0.28 (0.18, 0.37)**
All other purpose	**0.85 (<0.001)**	**2.26 (1.73, 2.79)**	**0.89 (<0.001)**	**0.75 (0.61, 0.89)**
All trials	**0.91 (<0.001)**	**19.29 (16.21, 22.36)**	**0.98 (<0.001)**	**0.81 (0.75, 0.88)**

*Note*: Bolded values indicate statistical significance at α = 0.05.

Abbreviation: CI, Confidence Interval.

### 3.4 COA use across landmark events

We focused on five regulatory guidelines and interest group recommendations relevant to the use of COAs in clinical trials (Figure 3A) and assessed changes in COA use before versus after each event for the subset of applicable trials. We noted a significant increase in COA use around every event considered (Table 3). To decouple the contribution of the individual events from the linear increase in COA use over the years, we considered regression models that included separate covariates for a given event, the initiation year, and the interaction between the two (Table 3; Figure S5A). In all these models the association between year and increase in likelihood of COA use across the relevant oncology study subset remained significant. Moreover, there were additional significant increases in the likelihood of PRO use among trials with a site in the EU before versus after 2005 (OR > 10.00, *p* = 0.007) corresponding to the EMA guidance for HRQoL. A similar change was noted for industry‐sponsored trials with a site in the US before versus after 2009 (OR > 10.00, *p* = 0.003) corresponding to the FDA Guidance for Industry PRO Use, and all interventional oncology trials before versus after 2013 (OR > 10.00, *p* < 0.001) corresponding to the ISOQOL PRO Recommendations, when accounting for the underlying increase in COA use over time and its interaction with the respective event years. As in oncology trials, the increase in COA use rates among non‐oncology trials across each event considered, as well as the association between year and COA use among every trial subset considered were significant. Compared to trends among oncology trials, PRO use rates among non‐oncology trials in the EU did not increase after 2005 (OR > 10.00, *p* = 0.187). However, PRO use as a primary or secondary outcome increased significantly after 2018 (OR > 10.00, *p* < 0.001) corresponding to SPIRIT‐PRO extension (Table 3; Figure S5B).

**TABLE 3:** Clinical outcomes assessment (COA) use across time among oncology trials. COA use across relevant dates.

Event	Year	Applicable trials^a^	Outcome	Conditions studied (*N* trials)	% COA use (*N*)			OR (*p*)			
					Before event	After event	Chi‐square (*p*)	Intercept	After event year	Year	Interaction: trial year & event year
EMA: general guidance for evaluation of HRQoL	2005	Trial site in the European Union	PRO use	Oncology (*N* = 9004)	8.9% (111/1242)	25.2% (1957/7762)	**159.38 (<0.001)**	0.00 (<0.001)	**261.06 (0.007)**	**1.23 (<0.001)**	**0.88 (0.007)**
				Non‐oncology (*N* = 64,581)	13.3% (744/ 5592)	24.4% (14,420/ 58,989)	**352.21 (<0.001)**	0.00 (<0.001)	52.86 (0.187)	**1.10 (<0.001)**	0.97 (0.187)
FDA guidance for industry PRO use	2009	Industry sponsor or collaborator; Trial site in US	PRO use	Oncology (*N* = 5975)	9.9% (221/2229)	15.8% (592/3746)	**40.725 (<0.001)**	0.00 (<0.001)	**203.97 (0.002)**	**1.15 (<0.001)**	**0.90 (0.002)**
				Non‐oncology (*N* = 39,588)	15.5% (2161/13,943)	21.3% (5475/25,645)	**198.20 (<0.001)**	0.00 (<0.001)	**134.43 (<0.001)**	**1.10 (<0.001)**	**0.94 (<0.001)**
CONSORT‐PRO extension	2013	Randomized trials	PRO as primary or secondary outcome	Oncology (*N* = 14,448)	14.7% (1075/7337)	27.3% (1941/7111)	**348.75 (<0.001)**	0.00 (<0.001)	31.63 (0.333)	**1.11 (<0.001)**	0.98 (0.333)
				Non‐oncology (*N* = 167,528)	15.5% (12,198/81,170)	23.1% (19,930/86,358)	**1749.10 (<0.001)**	0.00 (<0.001)	8.75 (0.386)	**1.06 (<0.001)**	1.00 (0.386)
ISOQOL PRO recommendations	2013	All	PRO use	Oncology (*N* = 35,415)	10.3% (1932/18,752)	20.3% (3379/16,663)	**687.97 (<0.001)**	0.00 (<0.001)	**130.87 (<0.001)**	**1.13 (<0.001)**	**0.94 (<0.001)**
				Non‐oncology (*N* = 244,440)	15.5% (18,334/118,101)	24.1% (30,409/126,339)	**2791.50 (<0.001)**	0.00 (<0.001)	**31.20 (<0.001)**	**1.07 (<0.001)**	**0.98 (<0.001)**
SPIRIT‐PRO extension	2018	All	PRO as primary or secondary outcome	Oncology (*N* = 35,415)	11.6% (3514/30,232)	20.3% (1052/5183)	**295.62 (<0.001)**	0.00 (<0.001)	193.00 (0.186)	**1.11 (<0.001)**	0.91 (0.186)
				Non‐oncology (*N* = 244,440)	15.8% (32,427/205,750)	22.8% (8836/38,690)	**1162.10 (<0.001)**	0.00 (<0.001)	**257.17 (<0.001)**	**1.07 (<0.001)**	**0.88 (<0.001)**

*Note*: Bolded values indicate statistical significance at α = 0.05.

Abbreviation: OR, Odds Ratio.

^a^ Out of all interventional oncology trials.

## 4 DISCUSSION

We assessed the landscape of clinical trials with a focus on understanding differences in COA incorporation distinct from traditional survival and response endpoints in oncology versus non‐oncology trials and the impact of regulatory and advocacy efforts to promote COA use in clinical research. COAs were used by 18% of oncology trials, lagging non‐oncology trials where use was reported in 26% of studies, albeit the latter rate represents use across diverse disease areas with previously reported heterogeneous landscape of COA use.^25^ Use of COAs by sponsor class was found to be similar for oncology trials, whereas for non‐oncology trials sponsorship of trials by NIH or other US federal agencies was associated with higher rates of COA use. Similar trends were found in associations between progressing phases, DMC use, randomization, and higher rates of COA use in both categories of trials. However, in contrast to non‐oncology trials, oncology trials focused on treatment had significantly lower rates of COA use compared to those focused on supportive care. The overall use of COAs increased over time across both oncology and non‐oncology trials, likely reflecting the cumulative impact of regulatory and advocacy efforts. Our assessment also found significant increases in COA use coinciding with focused guidelines, including the EMA guidance for HRQoL, the FDA Guidance for Industry PRO Use, and the ISOQOL PRO Recommendations but the magnitude of the impact could not be determined reliably, pointing to the need to assess additional factors underlying COA use trends.

Lower COA use rates in early phase clinical trials reiterate previously reported concerns about phase 1 cancer trials,^15^, ^26^ and likely reflect challenges of incorporating COA instruments into small, non‐randomized trials^11^ often assessing heterogeneous patient populations or multiple tumor types.^27^, ^28^ The importance of COAs in patient‐focused toxicity assessment^29^ and the drive to include medical benefit outcomes in early phase cancer trials^30^ call for incorporation of meaningful COA outcomes to supplement or strengthen early phase studies as advocated by groups including the NIH Office of Patient‐Centered Outcomes Research.^31^, ^32^ Guidelines targeting early‐phase COA use or adaptation of existing strategies for randomized trials may be beneficial.^9^, ^11^, ^12^

The growing role of COAs to establish net clinical benefit of novel therapies^33^ and impact regulatory decision making^25^, ^34^, ^35^ underscores the need to address the low COA use rates in treatment‐focused oncology trials. However, an important caveat when interpreting these rates is that they exclude the use of traditional survival and tumor response endpoints in oncology trials, therefore underestimating the use of ClinROs as they pertain to those endpoints. In fact, we estimate that 54% of oncology trials use these traditional endpoints (66% of treatment‐focused trials and 9% of supportive care trials), versus only 8% of non‐oncology trials (Table S7). The need to augment these traditional assessments for treatment‐focused trials is driven by the challenges of objectively assessing tumor response, resulting, for example, from inter‐tumor heterogeniety,^36^ clinical consequences of immune and targeted therapies that are distinct from traditional chemotherapy on which traditional radiographic criteria were developed,^37^, ^38^ and the need to establish that the assessed response translates into future benefit.^39^ Even among supportive care oncology trials, 51% did not use COAs despite emphasis on using COAs to justify the clinical benefit of non‐curative therapies.^33^ Moreover, as a recent assessment of the use of COAs in regulatory decision‐making discovered, patient experience as captured by COAs played a central role in FDA decisions when COAs were used as primary endpoints with uses in other endpoints primarily providing supporting information to contextualize rare or poorly‐characterized conditions.^35^ Our assessment found that among trials that reported COA use, only 23% of oncology trials versus 49% of non‐oncology trials used COAs as primary or co‐primary endpoints. Our results and prior reports^16^, ^40^ highlight the unmet recommendations for COA use in trials, which may contribute to the underrealized impact of COAs in drug development.

PROs were found to be the most frequently used category of COAs in both categories of trials (84% for oncology and 78% non‐oncology trials reporting COA use) tracking Eastern Research Group's recent assessment that 84% of FDA reviews for approved applications (submitted between 2017 and 2020) that mention patient experience data, mention PROs.^35^ The most frequently reported COA instruments in oncology trials reflected recommended instruments including the EORTC QLQ‐C30, reported by nearly one third of all oncology trials using COAs.^41^ However, our analysis also revealed a broad, heterogeneous group of COA instruments used in oncology compared to non‐oncology, with a third of the trials using generic instruments not specific to a disease group. Generic measures of quality of life that are more translatable across disease groups may be paired with measures of cancer site‐ and therapy‐specific efficacy and toxicity profiles, given the growing development of cancer‐specific therapies. Standardization of these efforts should reflect the FDA Patient‐Focused Drug Development guidelines^42^ and may be further facilitated by core outcome sets with specific COA recommendations within cancer types,^43^ cancer‐site‐specific extensions to validated COAs as in the EORTC QLQ, and instrument libraries such as the Patient‐Reported Outcomes Measurement Information System from which targeted items can be selected from a validated pool of questionnaires.^44^

Our study was limited to the search and analysis of data from the ClinicalTrials.gov registry, which was chosen for being the largest and among the more user‐friendly clinical trial registries.^45^ However, ClinicalTrials.gov, similarly to other registries, is marked by low quality of data reporting,^46^, ^47^ often lacking structured or standardized information, and often missing information on exploratory endpoints whose reporting in the registry remains optional. Similarly, use of the free‐access version of PROQOLID limited our search to a large but non‐exhaustive subset of curated COAs and which, for example, excludes global assessment scales and omits custom‐made instruments from industry or individual investigators. While these limitations have likely led to COA use estimates that are too conservative, it is reasonable to posit that oncology and non‐oncology trials are similarly affected, and that the differences between the two classes of trials are not artifacts of the search methodology. Among the more complex limitations, we recognize that while the cataloged instruments were curated to be valid and reliable, we did not have information on how reliably and appropriately they were used in the trials or whether they captured the experience of diverse participants. Event‐oriented analyses were limited by subjective selection of events, challenges in disambiguating the effect of individual events, and potential bias from increasing reporting quality over time in the registry.^16^ However, our programmatic approach enabled a comprehensive, replicable, and efficient review of the data from the registry and utilization of information from additional databases. Future analyses of this kind may benefit from a greater push for COA reporting standardization not only at the existing level of protocol‐building and publications,^10^, ^12^ but also for trial registries. Recommendations may include reporting of outcome placement of COAs, reporting specific instrument names, use of common terminologies within ClinicalTrials.gov, which, to the best of our knowledge, do not exist for COAs yet, and reporting of post‐hoc analyses where COA data from trials are published, if at all.^40^ The resulting data transparency may also incentivize more intentional, a priori planning of COA outcomes in trial design, and guide future iterations of similar evaluations of COA use trends to gauge the field's progress. These steps may be necessary in the ongoing efforts to establish COAs as integral parts of clinical trial outcomes and advance the state of the practice.

## AUTHOR CONTRIBUTIONS

**Yeonju Kim:** Conceptualization (equal); data curation (lead); formal analysis (lead); methodology (equal); visualization (lead); writing – original draft (equal); writing – review and editing (equal). **Mark R Gilbert:** Conceptualization (supporting); funding acquisition (equal); writing – review and editing (equal). **Terri S. Armstrong:** Conceptualization (supporting); funding acquisition (equal); writing – review and editing (equal). **Orieta Celiku:** Conceptualization (equal); data curation (supporting); formal analysis (supporting); methodology (equal); supervision (lead); visualization (supporting); writing – original draft (equal); writing – review and editing (equal).

## FUNDING INFORMATION

Intramural Research Program of the National Cancer Institute, National Institutes of Health.

## CONFLICT OF INTEREST STATEMENT

The authors declare no conflicts of interest.

## Supporting information


**Data S1.** Supporting Information

## Data Availability

The data were derived from the following resources available in the public domain: ClinicalTrials.gov (https://clinicaltrials.gov/), PROQOLID (https://eprovide.mapi‐trust.org/advanced‐search), MeSH (https://www.ncbi.nlm.nih.gov/mesh/).
