# Supplementary material for: Clinical outcome assessment trends in clinical trials—Contrasting oncology and non‐oncology trials
Source: Cancer Med. 2023 Jul 8;12(16):16945–57. doi: 10.1002/cam4.6325 (PMC10501237; doi:10.1002/cam4.6325)

**Supporting Information**

[Supporting Tables 1](#_Toc136327381)

[Supporting Table S1. 1](#_Toc136327382)

[Supporting Table S2. 7](#_Toc136327383)

[Supporting Table S3. 8](#_Toc136327384)

[Supporting Table S4. 9](#_Toc136327385)

[Supporting Table S5. 11](#_Toc136327386)

[Supporting Table S6. 13](#_Toc136327387)

[Supporting Table S7. 14](#_Toc136327388)

[Supporting Figures 15](#_Toc136327389)

[Supporting Figure S1. 15](#_Toc136327390)

[Supporting Figure S2. 16](#_Toc136327391)

[Supporting Figure S3. 17](#_Toc136327392)

[Supporting Figure S4. 18](#_Toc136327393)

[Supporting Figure S5. 19](#_Toc136327394)

# **Supporting Tables**

Supporting Table S1. Medical Subject Headings (MeSH) terms under “Neoplasms by Site” used to search for general oncology trials in the clinicaltrials.gov registry. *To exclude pediatric-targeted trials, we used the following parameter in our RPostgreSQL query: calculated_values.maximum_age_num <= 18.

| [**Neoplasms by Site [C04.588]**](https://meshb.nlm.nih.gov/record/ui?ui=D009371)   - - [Abdominal Neoplasms [C04.588.033]](https://meshb.nlm.nih.gov/record/ui?ui=D000008)     - [Peritoneal Neoplasms [C04.588.033.513]](https://meshb.nlm.nih.gov/record/ui?ui=D010534)     - [Retroperitoneal Neoplasms [C04.588.033.731]](https://meshb.nlm.nih.gov/record/ui?ui=D012186)     - [Sister Mary Joseph's Nodule [C04.588.033.740]](https://meshb.nlm.nih.gov/record/ui?ui=D058288)   - [Anal Gland Neoplasms [C04.588.083]](https://meshb.nlm.nih.gov/record/ui?ui=D000694)   - [Bone Neoplasms [C04.588.149]](https://meshb.nlm.nih.gov/record/ui?ui=D001859)     - [Adamantinoma [C04.588.149.030]](https://meshb.nlm.nih.gov/record/ui?ui=D050398)     - [Femoral Neoplasms [C04.588.149.276]](https://meshb.nlm.nih.gov/record/ui?ui=D005266)     - [Skull Neoplasms [C04.588.149.721]](https://meshb.nlm.nih.gov/record/ui?ui=D012888)       - [Jaw Neoplasms [C04.588.149.721.450]](https://meshb.nlm.nih.gov/record/ui?ui=D007573)         - [Mandibular Neoplasms [C04.588.149.721.450.583]](https://meshb.nlm.nih.gov/record/ui?ui=D008339)         - [Maxillary Neoplasms [C04.588.149.721.450.601]](https://meshb.nlm.nih.gov/record/ui?ui=D008441)         - [Palatal Neoplasms [C04.588.149.721.450.692]](https://meshb.nlm.nih.gov/record/ui?ui=D010157)       - [Nose Neoplasms [C04.588.149.721.600]](https://meshb.nlm.nih.gov/record/ui?ui=D009669)       - [Orbital Neoplasms [C04.588.149.721.656]](https://meshb.nlm.nih.gov/record/ui?ui=D009918)       - [Skull Base Neoplasms [C04.588.149.721.828]](https://meshb.nlm.nih.gov/record/ui?ui=D019292)     - [Spinal Neoplasms [C04.588.149.828]](https://meshb.nlm.nih.gov/record/ui?ui=D013125)   - [Breast Neoplasms [C04.588.180]](https://meshb.nlm.nih.gov/record/ui?ui=D001943)     - [Breast Carcinoma In Situ [C04.588.180.130]](https://meshb.nlm.nih.gov/record/ui?ui=D000071960)     - [Breast Neoplasms, Male [C04.588.180.260]](https://meshb.nlm.nih.gov/record/ui?ui=D018567)     - [Carcinoma, Ductal, Breast [C04.588.180.390]](https://meshb.nlm.nih.gov/record/ui?ui=D018270)     - [Carcinoma, Lobular [C04.588.180.437]](https://meshb.nlm.nih.gov/record/ui?ui=D018275)     - [Hereditary Breast and Ovarian Cancer Syndrome [C04.588.180.483]](https://meshb.nlm.nih.gov/record/ui?ui=D061325)     - [Inflammatory Breast Neoplasms [C04.588.180.576]](https://meshb.nlm.nih.gov/record/ui?ui=D058922)     - [Triple Negative Breast Neoplasms [C04.588.180.788]](https://meshb.nlm.nih.gov/record/ui?ui=D064726)     - [Unilateral Breast Neoplasms [C04.588.180.800]](https://meshb.nlm.nih.gov/record/ui?ui=D000069584)   - [Digestive System Neoplasms [C04.588.274]](https://meshb.nlm.nih.gov/record/ui?ui=D004067)     - [Biliary Tract Neoplasms [C04.588.274.120]](https://meshb.nlm.nih.gov/record/ui?ui=D001661)       - [Bile Duct Neoplasms [C04.588.274.120.250]](https://meshb.nlm.nih.gov/record/ui?ui=D001650)         - [Common Bile Duct Neoplasms [C04.588.274.120.250.250]](https://meshb.nlm.nih.gov/record/ui?ui=D003138)       - [Gallbladder Neoplasms [C04.588.274.120.401]](https://meshb.nlm.nih.gov/record/ui?ui=D005706)     - [Gastrointestinal Neoplasms [C04.588.274.476]](https://meshb.nlm.nih.gov/record/ui?ui=D005770)       - [Esophageal Neoplasms [C04.588.274.476.205]](https://meshb.nlm.nih.gov/record/ui?ui=D004938)         - [Esophageal Squamous Cell Carcinoma [C04.588.274.476.205.500]](https://meshb.nlm.nih.gov/record/ui?ui=D000077277)       - [Intestinal Neoplasms [C04.588.274.476.411]](https://meshb.nlm.nih.gov/record/ui?ui=D007414)         - [Cecal Neoplasms [C04.588.274.476.411.184]](https://meshb.nlm.nih.gov/record/ui?ui=D002430)   [Appendiceal Neoplasms [C04.588.274.476.411.184.290]](https://meshb.nlm.nih.gov/record/ui?ui=D001063)   - - - - - [Colorectal Neoplasms [C04.588.274.476.411.307]](https://meshb.nlm.nih.gov/record/ui?ui=D015179)   [Adenomatous Polyposis Coli [C04.588.274.476.411.307.089]](https://meshb.nlm.nih.gov/record/ui?ui=D011125)  [Gardner Syndrome [C04.588.274.476.411.307.089.393]](https://meshb.nlm.nih.gov/record/ui?ui=D005736)  [Colonic Neoplasms [C04.588.274.476.411.307.180]](https://meshb.nlm.nih.gov/record/ui?ui=D003110)  [Colitis-Associated Neoplasms [C04.588.274.476.411.307.180.400]](https://meshb.nlm.nih.gov/record/ui?ui=D000083023)  [Sigmoid Neoplasms [C04.588.274.476.411.307.180.800]](https://meshb.nlm.nih.gov/record/ui?ui=D012811)  [Colorectal Neoplasms, Hereditary Nonpolyposis [C04.588.274.476.411.307.190]](https://meshb.nlm.nih.gov/record/ui?ui=D003123)  [Rectal Neoplasms [C04.588.274.476.411.307.790]](https://meshb.nlm.nih.gov/record/ui?ui=D012004)  [Anus Neoplasms [C04.588.274.476.411.307.790.040]](https://meshb.nlm.nih.gov/record/ui?ui=D001005)  [Anal Gland Neoplasms [C04.588.274.476.411.307.790.040.040]](https://meshb.nlm.nih.gov/record/ui?ui=D000694)   - - - - - [Duodenal Neoplasms [C04.588.274.476.411.445]](https://meshb.nlm.nih.gov/record/ui?ui=D004379)         - [Ileal Neoplasms [C04.588.274.476.411.501]](https://meshb.nlm.nih.gov/record/ui?ui=D007078)         - [Jejunal Neoplasms [C04.588.274.476.411.523]](https://meshb.nlm.nih.gov/record/ui?ui=D007580)       - [Stomach Neoplasms [C04.588.274.476.767]](https://meshb.nlm.nih.gov/record/ui?ui=D013274)     - [Liver Neoplasms [C04.588.274.623]](https://meshb.nlm.nih.gov/record/ui?ui=D008113)       - [Adenoma, Liver Cell [C04.588.274.623.040]](https://meshb.nlm.nih.gov/record/ui?ui=D018248)       - [Carcinoma, Hepatocellular [C04.588.274.623.160]](https://meshb.nlm.nih.gov/record/ui?ui=D006528)       - [Liver Neoplasms, Experimental [C04.588.274.623.460]](https://meshb.nlm.nih.gov/record/ui?ui=D008114)     - [Pancreatic Neoplasms [C04.588.274.761]](https://meshb.nlm.nih.gov/record/ui?ui=D010190)       - [Adenoma, Islet Cell [C04.588.274.761.249]](https://meshb.nlm.nih.gov/record/ui?ui=D007516)         - [Insulinoma [C04.588.274.761.249.500]](https://meshb.nlm.nih.gov/record/ui?ui=D007340)       - [Carcinoma, Islet Cell [C04.588.274.761.500]](https://meshb.nlm.nih.gov/record/ui?ui=D018273)         - [Gastrinoma [C04.588.274.761.500.124]](https://meshb.nlm.nih.gov/record/ui?ui=D015408)         - [Glucagonoma [C04.588.274.761.500.249]](https://meshb.nlm.nih.gov/record/ui?ui=D005935)         - [Somatostatinoma [C04.588.274.761.500.500]](https://meshb.nlm.nih.gov/record/ui?ui=D013005)         - [Vipoma [C04.588.274.761.500.750]](https://meshb.nlm.nih.gov/record/ui?ui=D003969)       - [Carcinoma, Pancreatic Ductal [C04.588.274.761.750]](https://meshb.nlm.nih.gov/record/ui?ui=D021441)       - [Pancreatic Intraductal Neoplasms [C04.588.274.761.875]](https://meshb.nlm.nih.gov/record/ui?ui=D000077779)     - [Peritoneal Neoplasms [C04.588.274.780]](https://meshb.nlm.nih.gov/record/ui?ui=D010534)   - [Endocrine Gland Neoplasms [C04.588.322]](https://meshb.nlm.nih.gov/record/ui?ui=D004701)     - [Adrenal Gland Neoplasms [C04.588.322.078]](https://meshb.nlm.nih.gov/record/ui?ui=D000310)       - [Adrenal Cortex Neoplasms [C04.588.322.078.265]](https://meshb.nlm.nih.gov/record/ui?ui=D000306)     - [Multiple Endocrine Neoplasia [C04.588.322.400]](https://meshb.nlm.nih.gov/record/ui?ui=D009377)       - [Multiple Endocrine Neoplasia Type 1 [C04.588.322.400.500]](https://meshb.nlm.nih.gov/record/ui?ui=D018761)       - [Multiple Endocrine Neoplasia Type 2a [C04.588.322.400.505]](https://meshb.nlm.nih.gov/record/ui?ui=D018813)       - [Multiple Endocrine Neoplasia Type 2b [C04.588.322.400.510]](https://meshb.nlm.nih.gov/record/ui?ui=D018814)     - [Ovarian Neoplasms [C04.588.322.455]](https://meshb.nlm.nih.gov/record/ui?ui=D010051)       - [Carcinoma, Ovarian Epithelial [C04.588.322.455.199]](https://meshb.nlm.nih.gov/record/ui?ui=D000077216)       - [Granulosa Cell Tumor [C04.588.322.455.398]](https://meshb.nlm.nih.gov/record/ui?ui=D006106)       - [Hereditary Breast and Ovarian Cancer Syndrome [C04.588.322.455.431]](https://meshb.nlm.nih.gov/record/ui?ui=D061325)       - [Luteoma [C04.588.322.455.464]](https://meshb.nlm.nih.gov/record/ui?ui=D018311)       - [Meigs Syndrome [C04.588.322.455.531]](https://meshb.nlm.nih.gov/record/ui?ui=D008539)       - [Sertoli-Leydig Cell Tumor [C04.588.322.455.648]](https://meshb.nlm.nih.gov/record/ui?ui=D018310)       - [Thecoma [C04.588.322.455.765]](https://meshb.nlm.nih.gov/record/ui?ui=D013798)     - [Pancreatic Neoplasms [C04.588.322.475]](https://meshb.nlm.nih.gov/record/ui?ui=D010190)       - [Adenoma, Islet Cell [C04.588.322.475.249]](https://meshb.nlm.nih.gov/record/ui?ui=D007516)         - [Insulinoma [C04.588.322.475.249.500]](https://meshb.nlm.nih.gov/record/ui?ui=D007340)       - [Carcinoma, Islet Cell [C04.588.322.475.500]](https://meshb.nlm.nih.gov/record/ui?ui=D018273)         - [Gastrinoma [C04.588.322.475.500.124]](https://meshb.nlm.nih.gov/record/ui?ui=D015408)         - [Glucagonoma [C04.588.322.475.500.249]](https://meshb.nlm.nih.gov/record/ui?ui=D005935)         - [Somatostatinoma [C04.588.322.475.500.500]](https://meshb.nlm.nih.gov/record/ui?ui=D013005)         - [Vipoma [C04.588.322.475.500.750]](https://meshb.nlm.nih.gov/record/ui?ui=D003969)       - [Carcinoma, Pancreatic Ductal [C04.588.322.475.750]](https://meshb.nlm.nih.gov/record/ui?ui=D021441)       - [Pancreatic Intraductal Neoplasms [C04.588.322.475.875]](https://meshb.nlm.nih.gov/record/ui?ui=D000077779)     - [Paraneoplastic Endocrine Syndromes [C04.588.322.490]](https://meshb.nlm.nih.gov/record/ui?ui=D009384)     - [Parathyroid Neoplasms [C04.588.322.525]](https://meshb.nlm.nih.gov/record/ui?ui=D010282)     - [Pituitary Neoplasms [C04.588.322.609]](https://meshb.nlm.nih.gov/record/ui?ui=D010911)       - [ACTH-Secreting Pituitary Adenoma [C04.588.322.609.145]](https://meshb.nlm.nih.gov/record/ui?ui=D049913)         - [Nelson Syndrome [C04.588.322.609.145.500]](https://meshb.nlm.nih.gov/record/ui?ui=D009347)       - [Growth Hormone-Secreting Pituitary Adenoma [C04.588.322.609.292]](https://meshb.nlm.nih.gov/record/ui?ui=D049912)       - [Prolactinoma [C04.588.322.609.792]](https://meshb.nlm.nih.gov/record/ui?ui=D015175)     - [Testicular Neoplasms [C04.588.322.762]](https://meshb.nlm.nih.gov/record/ui?ui=D013736)       - [Sertoli-Leydig Cell Tumor [C04.588.322.762.500]](https://meshb.nlm.nih.gov/record/ui?ui=D018310)         - [Leydig Cell Tumor [C04.588.322.762.500.249]](https://meshb.nlm.nih.gov/record/ui?ui=D007984)         - [Sertoli Cell Tumor [C04.588.322.762.500.500]](https://meshb.nlm.nih.gov/record/ui?ui=D012707)     - [Thyroid Neoplasms [C04.588.322.894]](https://meshb.nlm.nih.gov/record/ui?ui=D013964)       - [Thyroid Cancer, Papillary [C04.588.322.894.400]](https://meshb.nlm.nih.gov/record/ui?ui=D000077273)       - [Thyroid Nodule [C04.588.322.894.800]](https://meshb.nlm.nih.gov/record/ui?ui=D016606)   - [Eye Neoplasms [C04.588.364]](https://meshb.nlm.nih.gov/record/ui?ui=D005134)     - [Conjunctival Neoplasms [C04.588.364.235]](https://meshb.nlm.nih.gov/record/ui?ui=D003230)     - [Intraocular Lymphoma [C04.588.364.447]](https://meshb.nlm.nih.gov/record/ui?ui=D064090)     - [Orbital Neoplasms [C04.588.364.659]](https://meshb.nlm.nih.gov/record/ui?ui=D009918)     - [Paraneoplastic Syndromes, Ocular [C04.588.364.738]](https://meshb.nlm.nih.gov/record/ui?ui=D059545)     - [Retinal Neoplasms [C04.588.364.818]](https://meshb.nlm.nih.gov/record/ui?ui=D019572)       - [Retinoblastoma [C04.588.364.818.760]](https://meshb.nlm.nih.gov/record/ui?ui=D012175)     - [Uveal Neoplasms [C04.588.364.978]](https://meshb.nlm.nih.gov/record/ui?ui=D014604)       - [Choroid Neoplasms [C04.588.364.978.223]](https://meshb.nlm.nih.gov/record/ui?ui=D002830)       - [Iris Neoplasms [C04.588.364.978.400]](https://meshb.nlm.nih.gov/record/ui?ui=D015811)   - [Head and Neck Neoplasms [C04.588.443]](https://meshb.nlm.nih.gov/record/ui?ui=D006258)     - [Squamous Cell Carcinoma of Head and Neck [C04.588.443.177]](https://meshb.nlm.nih.gov/record/ui?ui=D000077195)     - [Esophageal Neoplasms [C04.588.443.353]](https://meshb.nlm.nih.gov/record/ui?ui=D004938)       - [Esophageal Squamous Cell Carcinoma [C04.588.443.353.500]](https://meshb.nlm.nih.gov/record/ui?ui=D000077277)     - [Facial Neoplasms [C04.588.443.392]](https://meshb.nlm.nih.gov/record/ui?ui=D005153)       - [Eyelid Neoplasms [C04.588.443.392.500]](https://meshb.nlm.nih.gov/record/ui?ui=D005142)     - [Mouth Neoplasms [C04.588.443.591]](https://meshb.nlm.nih.gov/record/ui?ui=D009062)       - [Gingival Neoplasms [C04.588.443.591.402]](https://meshb.nlm.nih.gov/record/ui?ui=D005887)       - [Leukoplakia, Oral [C04.588.443.591.545]](https://meshb.nlm.nih.gov/record/ui?ui=D007972)         - [Leukoplakia, Hairy [C04.588.443.591.545.500]](https://meshb.nlm.nih.gov/record/ui?ui=D017733)       - [Lip Neoplasms [C04.588.443.591.550]](https://meshb.nlm.nih.gov/record/ui?ui=D008048)       - [Palatal Neoplasms [C04.588.443.591.692]](https://meshb.nlm.nih.gov/record/ui?ui=D010157)       - [Salivary Gland Neoplasms [C04.588.443.591.824]](https://meshb.nlm.nih.gov/record/ui?ui=D012468)         - [Parotid Neoplasms [C04.588.443.591.824.695]](https://meshb.nlm.nih.gov/record/ui?ui=D010307)         - [Sublingual Gland Neoplasms [C04.588.443.591.824.882]](https://meshb.nlm.nih.gov/record/ui?ui=D013362)         - [Submandibular Gland Neoplasms [C04.588.443.591.824.885]](https://meshb.nlm.nih.gov/record/ui?ui=D013365)       - [Tongue Neoplasms [C04.588.443.591.925]](https://meshb.nlm.nih.gov/record/ui?ui=D014062)     - [Otorhinolaryngologic Neoplasms [C04.588.443.665]](https://meshb.nlm.nih.gov/record/ui?ui=D010039)       - [Ear Neoplasms [C04.588.443.665.312]](https://meshb.nlm.nih.gov/record/ui?ui=D004428)       - [Laryngeal Neoplasms [C04.588.443.665.481]](https://meshb.nlm.nih.gov/record/ui?ui=D007822)       - [Nose Neoplasms [C04.588.443.665.650]](https://meshb.nlm.nih.gov/record/ui?ui=D009669)         - [Paranasal Sinus Neoplasms [C04.588.443.665.650.693]](https://meshb.nlm.nih.gov/record/ui?ui=D010255)       - [Pharyngeal Neoplasms [C04.588.443.665.710]](https://meshb.nlm.nih.gov/record/ui?ui=D010610)         - [Hypopharyngeal Neoplasms [C04.588.443.665.710.485]](https://meshb.nlm.nih.gov/record/ui?ui=D007012)         - [Nasopharyngeal Neoplasms [C04.588.443.665.710.650]](https://meshb.nlm.nih.gov/record/ui?ui=D009303)   [Nasopharyngeal Carcinoma [C04.588.443.665.710.650.500]](https://meshb.nlm.nih.gov/record/ui?ui=D000077274)   - - - - - [Oropharyngeal Neoplasms [C04.588.443.665.710.684]](https://meshb.nlm.nih.gov/record/ui?ui=D009959)   [Tonsillar Neoplasms [C04.588.443.665.710.684.800]](https://meshb.nlm.nih.gov/record/ui?ui=D014067)   - - - [Parathyroid Neoplasms [C04.588.443.680]](https://meshb.nlm.nih.gov/record/ui?ui=D010282)     - [Thyroid Neoplasms [C04.588.443.915]](https://meshb.nlm.nih.gov/record/ui?ui=D013964)       - [Thyroid Cancer, Papillary [C04.588.443.915.400]](https://meshb.nlm.nih.gov/record/ui?ui=D000077273)       - [Thyroid Nodule [C04.588.443.915.800]](https://meshb.nlm.nih.gov/record/ui?ui=D016606)     - [Tracheal Neoplasms [C04.588.443.925]](https://meshb.nlm.nih.gov/record/ui?ui=D014134)   - [Hematologic Neoplasms [C04.588.448]](https://meshb.nlm.nih.gov/record/ui?ui=D019337)     - [Bone Marrow Neoplasms [C04.588.448.200]](https://meshb.nlm.nih.gov/record/ui?ui=D019046)       - [Polycythemia Vera [C04.588.448.200.500]](https://meshb.nlm.nih.gov/record/ui?ui=D011087)   - [Mammary Neoplasms, Animal [C04.588.531]](https://meshb.nlm.nih.gov/record/ui?ui=D015674)     - [Mammary Neoplasms, Experimental [C04.588.531.500]](https://meshb.nlm.nih.gov/record/ui?ui=D008325)   - [Nervous System Neoplasms [C04.588.614]](https://meshb.nlm.nih.gov/record/ui?ui=D009423)     - [Central Nervous System Neoplasms [C04.588.614.250]](https://meshb.nlm.nih.gov/record/ui?ui=D016543)       - [Brain Neoplasms [C04.588.614.250.195]](https://meshb.nlm.nih.gov/record/ui?ui=D001932)         - [Cerebral Ventricle Neoplasms [C04.588.614.250.195.205]](https://meshb.nlm.nih.gov/record/ui?ui=D002551)   [Choroid Plexus Neoplasms [C04.588.614.250.195.205.200]](https://meshb.nlm.nih.gov/record/ui?ui=D016545)  [Papilloma, Choroid Plexus [C04.588.614.250.195.205.200.500]](https://meshb.nlm.nih.gov/record/ui?ui=D020288)   - - - - - [Infratentorial Neoplasms [C04.588.614.250.195.411]](https://meshb.nlm.nih.gov/record/ui?ui=D015192)   [Brain Stem Neoplasms [C04.588.614.250.195.411.100]](https://meshb.nlm.nih.gov/record/ui?ui=D020295)  [Diffuse Intrinsic Pontine Glioma [C04.588.614.250.195.411.100.500]](https://meshb.nlm.nih.gov/record/ui?ui=D000080443)  [Cerebellar Neoplasms [C04.588.614.250.195.411.211]](https://meshb.nlm.nih.gov/record/ui?ui=D002528)   - - - - - [Neurocytoma [C04.588.614.250.195.648]](https://meshb.nlm.nih.gov/record/ui?ui=D018306)         - [Pinealoma [C04.588.614.250.195.766]](https://meshb.nlm.nih.gov/record/ui?ui=D010871)         - [Supratentorial Neoplasms [C04.588.614.250.195.885]](https://meshb.nlm.nih.gov/record/ui?ui=D015173)   [Hypothalamic Neoplasms [C04.588.614.250.195.885.500]](https://meshb.nlm.nih.gov/record/ui?ui=D007029)  [Pallister-Hall Syndrome [C04.588.614.250.195.885.500.299]](https://meshb.nlm.nih.gov/record/ui?ui=D054975)  [Pituitary Neoplasms [C04.588.614.250.195.885.500.600]](https://meshb.nlm.nih.gov/record/ui?ui=D010911)   - - - - [Central Nervous System Cysts [C04.588.614.250.387]](https://meshb.nlm.nih.gov/record/ui?ui=D020863)         - [Arachnoid Cysts [C04.588.614.250.387.100]](https://meshb.nlm.nih.gov/record/ui?ui=D016080)         - [Colloid Cysts [C04.588.614.250.387.200]](https://meshb.nlm.nih.gov/record/ui?ui=D056364)       - [Meningeal Neoplasms [C04.588.614.250.580]](https://meshb.nlm.nih.gov/record/ui?ui=D008577)         - [Meningeal Carcinomatosis [C04.588.614.250.580.150]](https://meshb.nlm.nih.gov/record/ui?ui=D055756)         - [Meningioma [C04.588.614.250.580.500]](https://meshb.nlm.nih.gov/record/ui?ui=D008579)       - [Spinal Cord Neoplasms [C04.588.614.250.803]](https://meshb.nlm.nih.gov/record/ui?ui=D013120)         - [Epidural Neoplasms [C04.588.614.250.803.342]](https://meshb.nlm.nih.gov/record/ui?ui=D015174)     - [Cranial Nerve Neoplasms [C04.588.614.300]](https://meshb.nlm.nih.gov/record/ui?ui=D003390)       - [Neuroma, Acoustic [C04.588.614.300.015]](https://meshb.nlm.nih.gov/record/ui?ui=D009464)       - [Optic Nerve Neoplasms [C04.588.614.300.600]](https://meshb.nlm.nih.gov/record/ui?ui=D019574)         - [Optic Nerve Glioma [C04.588.614.300.600.600]](https://meshb.nlm.nih.gov/record/ui?ui=D020339)     - [Paraneoplastic Syndromes, Nervous System [C04.588.614.550]](https://meshb.nlm.nih.gov/record/ui?ui=D020361)       - [Anti-N-Methyl-D-Aspartate Receptor Encephalitis [C04.588.614.550.112]](https://meshb.nlm.nih.gov/record/ui?ui=D060426)       - [Limbic Encephalitis [C04.588.614.550.450]](https://meshb.nlm.nih.gov/record/ui?ui=D020363)       - [Myasthenia Gravis [C04.588.614.550.500]](https://meshb.nlm.nih.gov/record/ui?ui=D009157)         - [Lambert-Eaton Myasthenic Syndrome [C04.588.614.550.500.225]](https://meshb.nlm.nih.gov/record/ui?ui=D015624)       - [Myelitis, Transverse [C04.588.614.550.550]](https://meshb.nlm.nih.gov/record/ui?ui=D009188)       - [Opsoclonus-Myoclonus Syndrome [C04.588.614.550.600]](https://meshb.nlm.nih.gov/record/ui?ui=D053578)       - [Paraneoplastic Cerebellar Degeneration [C04.588.614.550.650]](https://meshb.nlm.nih.gov/record/ui?ui=D020362)       - [Paraneoplastic Polyneuropathy [C04.588.614.550.700]](https://meshb.nlm.nih.gov/record/ui?ui=D020364)     - [Peripheral Nervous System Neoplasms [C04.588.614.596]](https://meshb.nlm.nih.gov/record/ui?ui=D010524)       - [Cranial Nerve Neoplasms [C04.588.614.596.240]](https://meshb.nlm.nih.gov/record/ui?ui=D003390)         - [Neuroma, Acoustic [C04.588.614.596.240.015]](https://meshb.nlm.nih.gov/record/ui?ui=D009464)         - [Optic Nerve Neoplasms [C04.588.614.596.240.240]](https://meshb.nlm.nih.gov/record/ui?ui=D019574)   [Optic Nerve Glioma [C04.588.614.596.240.240.500]](https://meshb.nlm.nih.gov/record/ui?ui=D020339)   - - [Pelvic Neoplasms [C04.588.699]](https://meshb.nlm.nih.gov/record/ui?ui=D010386)   - [Skin Neoplasms [C04.588.805]](https://meshb.nlm.nih.gov/record/ui?ui=D012878)     - [Acanthoma [C04.588.805.040]](https://meshb.nlm.nih.gov/record/ui?ui=D049309)     - [Mastocytosis, Cutaneous [C04.588.805.309]](https://meshb.nlm.nih.gov/record/ui?ui=D034701)       - [Mastocytoma, Skin [C04.588.805.309.500]](https://meshb.nlm.nih.gov/record/ui?ui=D054705)       - [Urticaria Pigmentosa [C04.588.805.309.850]](https://meshb.nlm.nih.gov/record/ui?ui=D014582)     - [Sebaceous Gland Neoplasms [C04.588.805.578]](https://meshb.nlm.nih.gov/record/ui?ui=D012626)       - [Muir-Torre Syndrome [C04.588.805.578.500]](https://meshb.nlm.nih.gov/record/ui?ui=D055653)     - [Sweat Gland Neoplasms [C04.588.805.776]](https://meshb.nlm.nih.gov/record/ui?ui=D013544)   - [Soft Tissue Neoplasms [C04.588.839]](https://meshb.nlm.nih.gov/record/ui?ui=D012983)     - [Muscle Neoplasms [C04.588.839.500]](https://meshb.nlm.nih.gov/record/ui?ui=D019042)     - [Vascular Neoplasms [C04.588.839.750]](https://meshb.nlm.nih.gov/record/ui?ui=D019043)   - [Splenic Neoplasms [C04.588.842]](https://meshb.nlm.nih.gov/record/ui?ui=D013160)   - [Thoracic Neoplasms [C04.588.894]](https://meshb.nlm.nih.gov/record/ui?ui=D013899)     - [Heart Neoplasms [C04.588.894.309]](https://meshb.nlm.nih.gov/record/ui?ui=D006338)       - [Cardiac Papillary Fibroelastoma [C04.588.894.309.250]](https://meshb.nlm.nih.gov/record/ui?ui=D000084122)       - [Carney Complex [C04.588.894.309.500]](https://meshb.nlm.nih.gov/record/ui?ui=D056733)     - [Mediastinal Neoplasms [C04.588.894.479]](https://meshb.nlm.nih.gov/record/ui?ui=D008479)     - [Respiratory Tract Neoplasms [C04.588.894.797]](https://meshb.nlm.nih.gov/record/ui?ui=D012142)       - [Lung Neoplasms [C04.588.894.797.520]](https://meshb.nlm.nih.gov/record/ui?ui=D008175)         - [Adenocarcinoma of Lung [C04.588.894.797.520.055]](https://meshb.nlm.nih.gov/record/ui?ui=D000077192)   [Adenocarcinoma, Bronchiolo-Alveolar [C04.588.894.797.520.055.500]](https://meshb.nlm.nih.gov/record/ui?ui=D002282)   - - - - - [Bronchial Neoplasms [C04.588.894.797.520.109]](https://meshb.nlm.nih.gov/record/ui?ui=D001984)   [Carcinoma, Bronchogenic [C04.588.894.797.520.109.220]](https://meshb.nlm.nih.gov/record/ui?ui=D002283)  [Carcinoma, Non-Small-Cell Lung [C04.588.894.797.520.109.220.249]](https://meshb.nlm.nih.gov/record/ui?ui=D002289)  [Small Cell Lung Carcinoma [C04.588.894.797.520.109.220.624]](https://meshb.nlm.nih.gov/record/ui?ui=D055752)   - - - - - [Mesothelioma, Malignant [C04.588.894.797.520.173]](https://meshb.nlm.nih.gov/record/ui?ui=D000086002)         - [Multiple Pulmonary Nodules [C04.588.894.797.520.237]](https://meshb.nlm.nih.gov/record/ui?ui=D055613)         - [Pancoast Syndrome [C04.588.894.797.520.734]](https://meshb.nlm.nih.gov/record/ui?ui=D010178)         - [Pulmonary Blastoma [C04.588.894.797.520.867]](https://meshb.nlm.nih.gov/record/ui?ui=D018202)         - [Pulmonary Sclerosing Hemangioma [C04.588.894.797.520.933]](https://meshb.nlm.nih.gov/record/ui?ui=D047868)       - [Pleural Neoplasms [C04.588.894.797.640]](https://meshb.nlm.nih.gov/record/ui?ui=D010997)         - [Mesothelioma, Malignant [C04.588.894.797.640.350]](https://meshb.nlm.nih.gov/record/ui?ui=D000086002)         - [Pleural Effusion, Malignant [C04.588.894.797.640.700]](https://meshb.nlm.nih.gov/record/ui?ui=D016066)         - [Solitary Fibrous Tumor, Pleural [C04.588.894.797.640.800]](https://meshb.nlm.nih.gov/record/ui?ui=D054363)       - [Tracheal Neoplasms [C04.588.894.797.760]](https://meshb.nlm.nih.gov/record/ui?ui=D014134)     - [Thymus Neoplasms [C04.588.894.949]](https://meshb.nlm.nih.gov/record/ui?ui=D013953)       - [Thymoma [C04.588.894.949.500]](https://meshb.nlm.nih.gov/record/ui?ui=D013945)   - [Urogenital Neoplasms [C04.588.945]](https://meshb.nlm.nih.gov/record/ui?ui=D014565)     - [Genital Neoplasms, Female [C04.588.945.418]](https://meshb.nlm.nih.gov/record/ui?ui=D005833)       - [Fallopian Tube Neoplasms [C04.588.945.418.365]](https://meshb.nlm.nih.gov/record/ui?ui=D005185)       - [Uterine Neoplasms [C04.588.945.418.948]](https://meshb.nlm.nih.gov/record/ui?ui=D014594)         - [Endometrial Neoplasms [C04.588.945.418.948.585]](https://meshb.nlm.nih.gov/record/ui?ui=D016889)   [Carcinoma, Endometrioid [C04.588.945.418.948.585.124]](https://meshb.nlm.nih.gov/record/ui?ui=D018269)   - - - - - [Uterine Cervical Neoplasms [C04.588.945.418.948.850]](https://meshb.nlm.nih.gov/record/ui?ui=D002583)       - [Vaginal Neoplasms [C04.588.945.418.955]](https://meshb.nlm.nih.gov/record/ui?ui=D014625)       - [Vulvar Neoplasms [C04.588.945.418.968]](https://meshb.nlm.nih.gov/record/ui?ui=D014846)     - [Genital Neoplasms, Male [C04.588.945.440]](https://meshb.nlm.nih.gov/record/ui?ui=D005834)       - [Penile Neoplasms [C04.588.945.440.715]](https://meshb.nlm.nih.gov/record/ui?ui=D010412)       - [Prostatic Neoplasms [C04.588.945.440.770]](https://meshb.nlm.nih.gov/record/ui?ui=D011471)         - [Prostatic Neoplasms, Castration-Resistant [C04.588.945.440.770.500]](https://meshb.nlm.nih.gov/record/ui?ui=D064129)       - [Testicular Neoplasms [C04.588.945.440.915]](https://meshb.nlm.nih.gov/record/ui?ui=D013736)         - [Sertoli-Leydig Cell Tumor [C04.588.945.440.915.500]](https://meshb.nlm.nih.gov/record/ui?ui=D018310)   [Leydig Cell Tumor [C04.588.945.440.915.500.249]](https://meshb.nlm.nih.gov/record/ui?ui=D007984)  [Sertoli Cell Tumor [C04.588.945.440.915.500.500]](https://meshb.nlm.nih.gov/record/ui?ui=D012707)   - - - [Urologic Neoplasms [C04.588.945.947]](https://meshb.nlm.nih.gov/record/ui?ui=D014571)       - [Kidney Neoplasms [C04.588.945.947.535]](https://meshb.nlm.nih.gov/record/ui?ui=D007680)         - [Carcinoma, Renal Cell [C04.588.945.947.535.160]](https://meshb.nlm.nih.gov/record/ui?ui=D002292)         - [Wilms Tumor [C04.588.945.947.535.585]](https://meshb.nlm.nih.gov/record/ui?ui=D009396)   [Denys-Drash Syndrome [C04.588.945.947.535.585.220]](https://meshb.nlm.nih.gov/record/ui?ui=D030321)  [WAGR Syndrome [C04.588.945.947.535.585.950]](https://meshb.nlm.nih.gov/record/ui?ui=D017624)   - - - - - [Nephroma, Mesoblastic [C04.588.945.947.535.790]](https://meshb.nlm.nih.gov/record/ui?ui=D018201)       - [Ureteral Neoplasms [C04.588.945.947.940]](https://meshb.nlm.nih.gov/record/ui?ui=D014516)       - [Urethral Neoplasms [C04.588.945.947.945]](https://meshb.nlm.nih.gov/record/ui?ui=D014523)       - [Urinary Bladder Neoplasms [C04.588.945.947.960]](https://meshb.nlm.nih.gov/record/ui?ui=D001749)     - [Venereal Tumors, Veterinary [C04.588.945.956]](https://meshb.nlm.nih.gov/record/ui?ui=D014685) |
| --- |

Supporting Table S2. Names of countries categorized as belonging in the European Union for trials in the registry (start date 1985-2020).

| "United Kingdom", "Austria", "Italy", "Belgium", "Latvia", "Bulgaria", "Lithuania", "Croatia", "Luxembourg", "Cyprus", "Malta", "Czechia", "Netherlands", "Denmark", "Poland", "Estonia", "Portugal", "Finland", "Romania", "France", "Slovakia", "Germany", "Slovenia", "Greece", "Spain", "Hungary", "Sweden", "Ireland" |
| --- |

Supporting Table S3. Baseline trial characteristics of interventional studies of oncology and non-oncology diagnoses.

| **Trial Characteristic** | | **Number of Trials (%)** | |
| --- | --- | --- | --- |
|  |  | **Oncology** | **Non-Oncology** |
| **Phase** |  | *N = 26828** | *N = 132379* |
|  | Early Phase 1 | 555 (2.07%) | 2756 (2.08%) |
|  | Phase 1 | 4806 (17.91%) | 28749 (21.72%) |
|  | Phase 1/Phase 2 | 2889 (10.77%) | 8355 (6.31%) |
|  | Phase 2 | 12745 (47.51%) | 33461 (25.28%) |
|  | Phase 2/Phase 3 | 580 (2.16%) | 4944 (3.73%) |
|  | Phase 3 | 4358 (16.24%) | 27711 (20.93%) |
|  | Phase 4 | 895 (3.34%) | 26403 (19.95%) |
| **Recruitment Status** |  | *N = 35415* | *N = 244440* |
|  | Completed | 15650 (44.19%) | 148853 (60.90%) |
| Active | Recruiting | 6691 (18.89%) | 30523 (12.49%) |
|  | Active, not recruiting | 3237 (9.14%) | 10624 (4.35%) |
|  | Not yet recruiting | 736 (2.08%) | 4702 (1.92%) |
|  | Enrolling by invitation | 132 (0.37%) | 1834 (0.75%) |
| Suspended/Terminated/Withdrawn | Suspended | 202 (0.57%) | 1037 (0.42%) |
|  | Terminated | 3670 (10.36%) | 15421 (6.31%) |
|  | Withdrawn | 1285 (3.63%) | 6867 (2.81%) |
| All Other | Unknown status | 3812 (10.76%) | 24579 (10.06%) |
| **FDA Regulated Drug/Device** |  | *N = 12861* | *N = 91120* |
|  | Non-Regulated | 7593 (59.04%) | 65705 (72.11%) |
|  | Regulated | 5268 (40.96%) | 25415 (27.89%) |
| **Has DMC**** |  | *N = 28011* | *N = 203047* |
|  | DMC Not Used | 12774 (45.60%) | 123719 (60.93%) |
|  | DMC Used | 15237 (54.40%) | 79328 (39.07%) |
| **Primary Sponsor** |  | *N = 35414* | *N = 244345* |
|  | Industry | 7606 (21.48%) | 69988 (28.64%) |
|  | NIH or US Federal | 1676 (4.73%) | 8613 (3.52%) |
|  | Other | 26132 (73.79%) | 165744 (67.83%) |
| **Primary Purpose** |  | *N = 35415* | *N = 244440* |
|  | Treatment | 27128 (76.60%) | 156225 (63.91%) |
|  | Supportive Care | 1922 (5.43%) | 10429 (4.27%) |
|  | Diagnostic | 2493 (7.04%) | 9915 (4.06%) |
|  | All Other | 3872 (10.93%) | 67871 (27.77%) |
| **Blinding (n = 2838)** |  | *N = 33929* | *N = 240886* |
|  | Blinded | 5419 (15.97%) | 114956 (47.72%) |
|  | Non-Blinded | 28510 (84.03%) | 125930 (52.28%) |
| **Number of Arms** |  | *N = 31341* | *N = 224791* |
|  | Multi-Arm | 16799 (53.60%) | 173763 (77.30%) |
|  | Single-Arm | 14542 (46.40%) | 51028 (22.70%) |
| **Randomization (n = 1287)** |  | *N = 33423* | *N = 238652* |
|  | Randomized | 14448 (43.23%) | 167528 (70.20%) |
|  | Non-Randomized | 18975 (56.77%) | 71124 (29.80%) |

*number of trials reporting trial characteristic; ** DMC = Data Monitoring Committee

Supporting Table S4. Top COA instruments used among interventional oncology trials, and subsets of trials with the primary purpose of treatment, supportive care, and diagnostic. N indicates the number of trials using the instrument and percent of trials using the instrument out of all trials in each category. COAs used by less than 4 trials (<1% of interventional trials) were excluded.

| **COA Name** | **N Trials Using COA** | | | | |  |
| --- | --- | --- | --- | --- | --- | --- |
|  | (% of trials reporting COAs) | | | | |  |
|  | **All Trials** | **Primary Purpose of Treatment** | **Primary Purpose of Supportive Care** | **Primary Purpose of Diagnostic** | **All Other Primary Purpose** | |
| **Any COA** | 6331 | 4648 | 946 | 144 | 593 | |
| **EORTC Quality of Life Questionnaire - Core Questionnaire (EORTC QLQ-C30)** | 1864 (29.44%) | 1522 (32.75%) | 185 (19.56%) | 20 (13.89%) | 137 (23.1%) | |
| **Eastern Cooperative Oncology Group Performance Status scale (ECOG Performance Status)** | 914 (14.44%) | 836 (17.99%) | 23 (2.43%) | 19 (13.19%) | 36 (6.07%) | |
| **Euroqol EQ-5D (EQ-5D)** | 616 (9.73%) | 494 (10.63%) | 55 (5.81%) | 16 (11.11%) | 51 (8.6%) | |
| **Hospital Anxiety and Depression Scale (HADS)** | 305 (4.82%) | 89 (1.91%) | 156 (16.49%) | 6 (4.17%) | 54 (9.11%) | |
| **Brief Pain Inventory (BPI)** | 284 (4.49%) | 194 (4.17%) | 64 (6.77%) | 2 (1.39%) | 24 (4.05%) | |
| **SF-36 Health Survey (SF-36® / SF-36v2®)** | 264 (4.17%) | 141 (3.03%) | 75 (7.93%) | 6 (4.17%) | 42 (7.08%) | |
| **EuroQoL 5-Dimension 5-Level (EQ-5D-5L)** | 242 (3.82%) | 194 (4.17%) | 24 (2.54%) | 9 (6.25%) | 15 (2.53%) | |
| **Functional Assessment of Cancer Therapy - General (FACT-G)** | 242 (3.82%) | 140 (3.01%) | 76 (8.03%) | 0 (0%) | 26 (4.38%) | |
| **Functional Assessment of Chronic Illness Therapy Measurement System (FACIT)** | 198 (3.13%) | 94 (2.02%) | 78 (8.25%) | 0 (0%) | 26 (4.38%) | |
| **Functional Assessment of Cancer Therapy - Breast Cancer (FACT-B)** | 194 (3.06%) | 117 (2.52%) | 54 (5.71%) | 0 (0%) | 23 (3.88%) | |
| **Karnofsky Performance Status (KPS)** | 192 (3.03%) | 171 (3.68%) | 13 (1.37%) | 3 (2.08%) | 5 (0.84%) | |
| **International Index of Erectile Function (IIEF)** | 183 (2.89%) | 137 (2.95%) | 17 (1.8%) | 8 (5.56%) | 21 (3.54%) | |
| **Functional Assessment of Cancer Therapy - Prostate Cancer (FACT-P)** | 157 (2.48%) | 126 (2.71%) | 23 (2.43%) | 0 (0%) | 8 (1.35%) | |
| **Expanded Prostate Cancer Index Composite (EPIC)** | 144 (2.27%) | 116 (2.5%) | 19 (2.01%) | 1 (0.69%) | 8 (1.35%) | |
| **Functional Assessment of Cancer Therapy - Lung Cancer (FACT-L)** | 136 (2.15%) | 108 (2.32%) | 22 (2.33%) | 1 (0.69%) | 5 (0.84%) | |
| **MD Anderson Symptom Inventory (MDASI)** | 131 (2.07%) | 91 (1.96%) | 32 (3.38%) | 1 (0.69%) | 7 (1.18%) | |
| **International Prostate Symptom Score (I-PSS)** | 123 (1.94%) | 103 (2.22%) | 6 (0.63%) | 5 (3.47%) | 9 (1.52%) | |
| **Quality of Life Scale (QLS)** | 105 (1.66%) | 76 (1.64%) | 20 (2.11%) | 0 (0%) | 9 (1.52%) | |
| **Quality of Life Scale (QOLS)** | 105 (1.66%) | 76 (1.64%) | 20 (2.11%) | 0 (0%) | 9 (1.52%) | |
| **Pittsburgh Sleep Quality Index (PSQI)** | 104 (1.64%) | 33 (0.71%) | 50 (5.29%) | 1 (0.69%) | 20 (3.37%) | |
| **EORTC Quality of Life Questionnaire - Lung Cancer Module (EORTC QLQ-LC13)** | 98 (1.55%) | 90 (1.94%) | 3 (0.32%) | 1 (0.69%) | 4 (0.67%) | |
| **International Index of Erectile Function - 5 items or Sexual Health Inventory for Men (IIEF-5 or SHIM)** | 95 (1.5%) | 76 (1.64%) | 7 (0.74%) | 6 (4.17%) | 6 (1.01%) | |
| **Brief Pain Inventory - Short form (BPI-SF)** | 93 (1.47%) | 78 (1.68%) | 10 (1.06%) | 0 (0%) | 5 (0.84%) | |
| **Instrumental Activities of Daily Living (IADL)** | 86 (1.36%) | 55 (1.18%) | 16 (1.69%) | 2 (1.39%) | 13 (2.19%) | |
| **Center for Epidemiologic Studies Depression Scale (CES-D)** | 84 (1.33%) | 24 (0.52%) | 51 (5.39%) | 1 (0.69%) | 8 (1.35%) | |
| **Lung Cancer Symptom Scale (LCSS)** | 84 (1.33%) | 75 (1.61%) | 6 (0.63%) | 1 (0.69%) | 2 (0.34%) | |
| **SF-12 Health Survey (SF-12® / SF-12v2®)** | 84 (1.33%) | 51 (1.1%) | 23 (2.43%) | 0 (0%) | 10 (1.69%) | |
| **Brief Fatigue Inventory (BFI)** | 83 (1.31%) | 42 (0.9%) | 34 (3.59%) | 2 (1.39%) | 5 (0.84%) | |
| **Functional Assessment of Chronic Illness Therapy - Fatigue (FACIT-F)** | 79 (1.25%) | 31 (0.67%) | 36 (3.81%) | 0 (0%) | 12 (2.02%) | |
| **Patient Health Questionnaire (PHQ)** | 76 (1.2%) | 22 (0.47%) | 46 (4.86%) | 0 (0%) | 8 (1.35%) | |
| **Assessment of Quality of Life (AQoL)** | 63 (1%) | 49 (1.05%) | 3 (0.32%) | 1 (0.69%) | 10 (1.69%) | |
| **EORTC Quality of Life Questionnaire - Breast Cancer Module (EORTC QLQ-BR23)** | 63 (1%) | 47 (1.01%) | 8 (0.85%) | 1 (0.69%) | 7 (1.18%) | |
| **Female Sexual Function Index (FSFI)** | 63 (1%) | 37 (0.8%) | 17 (1.8%) | 0 (0%) | 9 (1.52%) | |
| **Functional Assessment of Cancer Therapy - Ovarian Cancer (FACT-O)** | 63 (1%) | 50 (1.08%) | 11 (1.16%) | 0 (0%) | 2 (0.34%) | |
| **Profile of Mood States (POMS)** | 59 (0.93%) | 17 (0.37%) | 34 (3.59%) | 0 (0%) | 8 (1.35%) | |
| **EORTC Quality of Life Questionnaire - Brain Cancer Module (EORTC QLQ-BN20)** | 58 (0.92%) | 51 (1.1%) | 2 (0.21%) | 1 (0.69%) | 4 (0.67%) | |
| **6-Minute Walk Test (6-MWT or SMWT)** | 57 (0.9%) | 26 (0.56%) | 25 (2.64%) | 1 (0.69%) | 5 (0.84%) | |
| **Hopkins Verbal Learning Test (HVLT)** | 56 (0.88%) | 44 (0.95%) | 7 (0.74%) | 0 (0%) | 5 (0.84%) | |
| **Trail Making Test (TMT)** | 55 (0.87%) | 40 (0.86%) | 11 (1.16%) | 0 (0%) | 4 (0.67%) | |
| **Functional Assessment of Cancer Therapy - Hepatobiliary Cancer (FACT-Hep)** | 54 (0.85%) | 41 (0.88%) | 8 (0.85%) | 1 (0.69%) | 4 (0.67%) | |
| **Mini-Mental State Examination (MMSE®)** | 47 (0.74%) | 40 (0.86%) | 3 (0.32%) | 1 (0.69%) | 3 (0.51%) | |
| **Quantitative Myasthenia Gravis Score (QMG)** | 47 (0.74%) | 47 (1.01%) | 0 (0%) | 0 (0%) | 0 (0%) | |
| **Functional Assessment of Cancer Therapy-Colorectal cancer (FACT-C)** | 46 (0.73%) | 31 (0.67%) | 12 (1.27%) | 0 (0%) | 3 (0.51%) | |
| **Disability Rating Scale (DRS)** | 45 (0.71%) | 37 (0.8%) | 3 (0.32%) | 1 (0.69%) | 4 (0.67%) | |
| **EORTC Quality of life - Head and Neck Cancer Module (EORTC QLQ-H&N35)** | 45 (0.71%) | 33 (0.71%) | 7 (0.74%) | 1 (0.69%) | 4 (0.67%) | |
| **Functional Assessment of Chronic Illness Therapy - Fatigue Scale (FACIT-Fatigue)** | 39 (0.62%) | 19 (0.41%) | 11 (1.16%) | 0 (0%) | 9 (1.52%) | |
| **MyeloProliferative Neoplasm Symptom Assessment Form (MPN-SAF)** | 39 (0.62%) | 38 (0.82%) | 1 (0.11%) | 0 (0%) | 0 (0%) | |
| **BREAST-Q™ (BREAST-Q™)** | 38 (0.6%) | 26 (0.56%) | 6 (0.63%) | 0 (0%) | 6 (1.01%) | |
| **Elective Surgery Acuity Scale (ESAS)** | 38 (0.6%) | 15 (0.32%) | 19 (2.01%) | 2 (1.39%) | 2 (0.34%) | |
| **Functional Assessment Of Cancer Therapy - Brain (FACT-Br)** | 38 (0.6%) | 35 (0.75%) | 2 (0.21%) | 0 (0%) | 1 (0.17%) | |

Supporting Table S5. Top COA instruments used among interventional non-oncology trials, and subsets of trials with the primary purpose of treatment, supportive care, and diagnostic. N indicates the number of trials using the instrument and percent of trials using the instrument out of all trials in each category. COAs used by less than 4 trials (<1% of interventional trials) were excluded.

| **COA Name** | **N Trials Using COA** | | | | |
| --- | --- | --- | --- | --- | --- |
|  | (% of trials reporting COAs) | | | | |
|  | **All Trials** | **Primary Purpose of Treatment** | **Primary Purpose of Supportive Care** | **Primary Purpose of Diagnostic** | **All Other Primary Purpose** |
| **Any COA** | 62,544 | 46,126 | 3,854 | 1,117 | 11,447 |
| **SF-36 Health Survey (SF-36® / SF-36v2®)** | 4971 (7.95%) | 3886 (8.42%) | 318 (8.25%) | 49 (4.39%) | 718 (6.27%) |
| **Euroqol EQ-5D (EQ-5D)** | 3813 (6.1%) | 2876 (6.24%) | 243 (6.31%) | 69 (6.18%) | 625 (5.46%) |
| **Patient Health Questionnaire (PHQ)** | 2318 (3.71%) | 1400 (3.04%) | 278 (7.21%) | 24 (2.15%) | 616 (5.38%) |
| **Hospital Anxiety and Depression Scale (HADS)** | 2113 (3.38%) | 1312 (2.84%) | 354 (9.19%) | 42 (3.76%) | 405 (3.54%) |
| **Clinical Global Impressions scale - Improvement, Severity, Change and Efficacy (CGI-I, CGI-S, CGI-C and CGI-E)** | 1828 (2.92%) | 1751 (3.8%) | 9 (0.23%) | 5 (0.45%) | 63 (0.55%) |
| **Pittsburgh Sleep Quality Index (PSQI)** | 1616 (2.58%) | 1089 (2.36%) | 159 (4.13%) | 12 (1.07%) | 356 (3.11%) |
| **SF-12 Health Survey (SF-12® / SF-12v2®)** | 1463 (2.34%) | 980 (2.12%) | 125 (3.24%) | 20 (1.79%) | 338 (2.95%) |
| **Western Ontario and McMaster Universities Arthritis Index (WOMAC®)** | 1409 (2.25%) | 1235 (2.68%) | 49 (1.27%) | 8 (0.72%) | 117 (1.02%) |
| **Mini-Mental State Examination (MMSE®)** | 1385 (2.21%) | 947 (2.05%) | 88 (2.28%) | 48 (4.3%) | 302 (2.64%) |
| **Montgomery-Asberg Depression Rating Scale (MADRS)** | 1353 (2.16%) | 1200 (2.6%) | 28 (0.73%) | 13 (1.16%) | 112 (0.98%) |
| **EuroQoL 5-Dimension 5-Level (EQ-5D-5L)** | 1318 (2.11%) | 954 (2.07%) | 101 (2.62%) | 26 (2.33%) | 237 (2.07%) |
| **Brief Pain Inventory (BPI)** | 1264 (2.02%) | 979 (2.12%) | 118 (3.06%) | 12 (1.07%) | 155 (1.35%) |
| **Hamilton Depression Rating Scale (HAM-D)** | 1232 (1.97%) | 1091 (2.37%) | 19 (0.49%) | 15 (1.34%) | 107 (0.93%) |
| **Early Treatment of Diabetic Retinopathy Scale (ETDRS)** | 1109 (1.77%) | 996 (2.16%) | 10 (0.26%) | 17 (1.52%) | 86 (0.75%) |
| **Positive and Negative Syndrome Scale for Schizophrenia (PANSS)** | 1075 (1.72%) | 941 (2.04%) | 14 (0.36%) | 16 (1.43%) | 104 (0.91%) |
| **Health Assessment Questionnaire (HAQ)** | 1038 (1.66%) | 903 (1.96%) | 34 (0.88%) | 10 (0.9%) | 91 (0.79%) |
| **EORTC Quality of Life Questionnaire - Core Questionnaire (EORTC QLQ-C30)** | 1008 (1.61%) | 791 (1.71%) | 120 (3.11%) | 13 (1.16%) | 84 (0.73%) |
| **Quality of Life Scale (QLS)** | 996 (1.59%) | 736 (1.6%) | 117 (3.04%) | 4 (0.36%) | 139 (1.21%) |
| **Quality of Life Scale (QOLS)** | 996 (1.59%) | 736 (1.6%) | 117 (3.04%) | 4 (0.36%) | 139 (1.21%) |
| **Montreal Cognitive Assessment (MoCA)** | 994 (1.59%) | 635 (1.38%) | 66 (1.71%) | 22 (1.97%) | 271 (2.37%) |
| **Unified Parkinson’s Disease Rating Scale (UPDRS)** | 977 (1.56%) | 813 (1.76%) | 28 (0.73%) | 19 (1.7%) | 117 (1.02%) |
| **6-Minute Walk Test (6-MWT or SMWT)** | 957 (1.53%) | 706 (1.53%) | 77 (2%) | 27 (2.42%) | 147 (1.28%) |
| **Epworth Sleepiness Scale (ESS)** | 914 (1.46%) | 727 (1.58%) | 47 (1.22%) | 38 (3.4%) | 102 (0.89%) |
| **Trail Making Test (TMT)** | 877 (1.4%) | 563 (1.22%) | 58 (1.5%) | 18 (1.61%) | 238 (2.08%) |
| **Generalized Anxiety Disorder - 7 (GAD-7)** | 875 (1.4%) | 567 (1.23%) | 96 (2.49%) | 7 (0.63%) | 205 (1.79%) |
| **Center for Epidemiologic Studies Depression Scale (CES-D)** | 868 (1.39%) | 435 (0.94%) | 138 (3.58%) | 9 (0.81%) | 286 (2.5%) |
| **Modified Rankin Scale (MRS)** | 853 (1.36%) | 700 (1.52%) | 18 (0.47%) | 18 (1.61%) | 117 (1.02%) |
| **Oswestry Disability Index (ODI)** | 847 (1.35%) | 725 (1.57%) | 22 (0.57%) | 18 (1.61%) | 82 (0.72%) |
| **Timed Up & Go Test (TUG)** | 826 (1.32%) | 614 (1.33%) | 56 (1.45%) | 5 (0.45%) | 151 (1.32%) |
| **Barthel Index (Barthel Index)** | 801 (1.28%) | 643 (1.39%) | 45 (1.17%) | 5 (0.45%) | 108 (0.94%) |
| **Beck Depression Inventory® - Second Edition (BDI®-II)** | 779 (1.25%) | 621 (1.35%) | 50 (1.3%) | 3 (0.27%) | 105 (0.92%) |
| **Columbia-Suicide Severity Rating Scale (C-SSRS)** | 770 (1.23%) | 629 (1.36%) | 7 (0.18%) | 4 (0.36%) | 130 (1.14%) |
| **National Institutes of Health Stroke Scale (NIHSS)** | 758 (1.21%) | 639 (1.39%) | 12 (0.31%) | 18 (1.61%) | 89 (0.78%) |
| **Knee Injury and Osteoarthritis Outcome Score (KOOS)** | 738 (1.18%) | 619 (1.34%) | 32 (0.83%) | 5 (0.45%) | 82 (0.72%) |
| **Psoriasis Area and Severity Index (PASI)** | 733 (1.17%) | 680 (1.47%) | 10 (0.26%) | 5 (0.45%) | 38 (0.33%) |
| **Sequential Organ Failure Assessment (SOFA)** | 699 (1.12%) | 548 (1.19%) | 24 (0.62%) | 23 (2.06%) | 104 (0.91%) |
| **Instrumental Activities of Daily Living (IADL)** | 689 (1.1%) | 422 (0.91%) | 75 (1.95%) | 15 (1.34%) | 177 (1.55%) |
| **Insomnia Severity Index (ISI)** | 668 (1.07%) | 531 (1.15%) | 45 (1.17%) | 6 (0.54%) | 86 (0.75%) |
| **Ashworth Scale (Ashworth Scale)** | 655 (1.05%) | 568 (1.23%) | 27 (0.7%) | 6 (0.54%) | 54 (0.47%) |
| **Expanded Disability Status Scale (EDSS)** | 602 (0.96%) | 505 (1.09%) | 24 (0.62%) | 11 (0.98%) | 62 (0.54%) |
| **Berg Balance Scale (BBS)** | 592 (0.95%) | 449 (0.97%) | 42 (1.09%) | 8 (0.72%) | 93 (0.81%) |
| **Positive and Negative Affect Schedule (PANAS)** | 591 (0.94%) | 286 (0.62%) | 49 (1.27%) | 3 (0.27%) | 253 (2.21%) |
| **St George’s Respiratory Questionnaire (SGRQ)** | 589 (0.94%) | 497 (1.08%) | 31 (0.8%) | 4 (0.36%) | 57 (0.5%) |
| **Modified Ashworth Scale (MAS)** | 578 (0.92%) | 502 (1.09%) | 24 (0.62%) | 5 (0.45%) | 47 (0.41%) |
| **Eastern Cooperative Oncology Group Performance Status scale (ECOG Performance Status)** | 562 (0.9%) | 472 (1.02%) | 43 (1.12%) | 9 (0.81%) | 38 (0.33%) |
| **Profile of Mood States (POMS)** | 557 (0.89%) | 273 (0.59%) | 84 (2.18%) | 10 (0.9%) | 190 (1.66%) |
| **Beck Anxiety Inventory® (BAI®)** | 533 (0.85%) | 409 (0.89%) | 27 (0.7%) | 1 (0.09%) | 96 (0.84%) |
| **Neck Disability Index (NDI)** | 530 (0.85%) | 469 (1.02%) | 8 (0.21%) | 9 (0.81%) | 44 (0.38%) |
| **Functional Assessment of Chronic Illness Therapy Measurement System (FACIT)** | 527 (0.84%) | 377 (0.82%) | 105 (2.72%) | 3 (0.27%) | 42 (0.37%) |
| **Hamilton Anxiety Scale (HAM-A)** | 520 (0.83%) | 466 (1.01%) | 9 (0.23%) | 4 (0.36%) | 41 (0.36%) |

Supporting Table S6. COA use across time among non-oncology trials. Correlation analysis and linear regression between number and proportion of trials using COAs each year, stratified by COA type and primary purpose of trials.

|  | **N Trials Over Time** | | | **% of Trials Over Time** | | |
| --- | --- | --- | --- | --- | --- | --- |
|  | **R** (p) | **β** (trials per year; 95% CI^2^) | **R** (p) | | **β** (% of trials per year; 95% CI) |  |
| **COA Type** |  |  |  | |  |  |
| PRO | **0.90 (<0.00001)** | **146.40 (121.58, 171.23)** | **0.98 (<0.00001)** | | **0.78 (0.72, 0.83)** |  |
| ClinRO | **0.92 (<0.00001)** | **79.08 (67.11, 91.04)** | **0.97 (<0.00001)** | | **0.41 (0.38, 0.45)** |  |
| PerfO | **0.87 (<0.00001)** | **22.15 (17.79, 26.51)** | **0.95 (<0.00001)** | | **0.12 (0.11, 0.14)** |  |
| ObsRO | **0.87 (<0.00001)** | **26.24 (21.06, 31.42)** | **0.95 (<0.00001)** | | **0.14 (0.12, 0.16)** |  |
| Composite | **0.87 (<0.00001)** | **30.98 (24.89, 37.07)** | **0.95 (<0.00001)** | | **0.18 (0.16, 0.20)** |  |
| **Primary Purpose** |  |  |  | |  |  |
| Treatment | **0.93 (<0.00001)** | **132.00 (113.09, 150.82)** | **0.99 (<0.00001)** | | **1.18 (1.11, 1.26)** |  |
| Supportive Care | **0.87 (<0.00001)** | **17.07 (13.09, 21.04)** | **0.95 (<0.00001)** | | **1.52 (1.32, 1.72)** |  |
| Diagnostic | **0.90 (<0.00001)** | **4.43 (3.58, 5.27)** | **0.69 (0.00004)** | | **0.43 (0.25, 0.61)** |  |
| All Other Purpose | **0.85 (<0.00001)** | **36.90 (28.92, 44.88)** | **0.92 (<0.00001)** | | **0.64 (0.55, 0.73)** |  |
| **All Trials** | **0.91 (<0.00001)** | **185.25 (155.30, 215.20)** | **0.98 (<0.00001)** | | **0.94 (0.87, 1.02)** |  |

Bolded values indicate statistical significance at α = 0.05; 1. Percent using COAs out of all trials in respective category; 2. CI=Confidence Interval.

Supporting Table S7. Rates of use of overall survival (OS), progression free survival (PFS) and response as trial endpoints and comparison with the rates of use of COAs as identified by search of individual instruments.

| **Disease area** | **Primary purpose** | **N trials (% out of all trials in category/row)** | | | | | |
| --- | --- | --- | --- | --- | --- | --- | --- |
|  |  | **OS** | **PFS** | **response** | **COAs** | **OS/PFS/response** | **any** |
| **Oncology** | **All** | 12950 (37%) | 12641 (36%) | 12800 (36%) | 6360 (18%) | 18949 (54%) | 21825 (62%) |
|  | Treatment | 12366 (46%) | 12172 (45%) | 12402 (46%) | 4663 (17%) | 17962 (66%) | 19384 (71%) |
|  | Supportive care | 105 (5%) | 69 (4%) | 75 (4%) | 948 (49%) | 178 (9%) | 1037 (54%) |
|  | Diagnostic | 142 (6%) | 148 (6%) | 99 (4%) | 151 (6%) | 270 (11%) | 395 (16%) |
|  | Other | 337 (9%) | 252 (7%) | 224 (6%) | 598 (15%) | 539 (14%) | 1009 (26%) |
| **Non-Oncology** | **All** | 9180 (4%) | 8516 (3%) | 14116 (6%) | 62672 (26%) | 18836 (8%) | 77668 (32%) |
|  | Treatment | 8612 (6%) | 8188 (5%) | 12881 (8%) | 46186 (30%) | 17007 (11%) | 59785 (38%) |
|  | Supportive care | 108 (1%) | 44 (<0.5%) | 163 (2%) | 3862 (37%) | 272 (3%) | 4018 (39%) |
|  | Diagnostic | 81 (1%) | 80 (1%) | 94 (1%) | 1123 (11%) | 197 (2%) | 1292 (13%) |
|  | Other | 379 (1%) | 204 (<0.5%) | 978 (1%) | 11501 (17%) | 1360 (2%) | 12573 (19%) |

# **Supporting Figures**

**
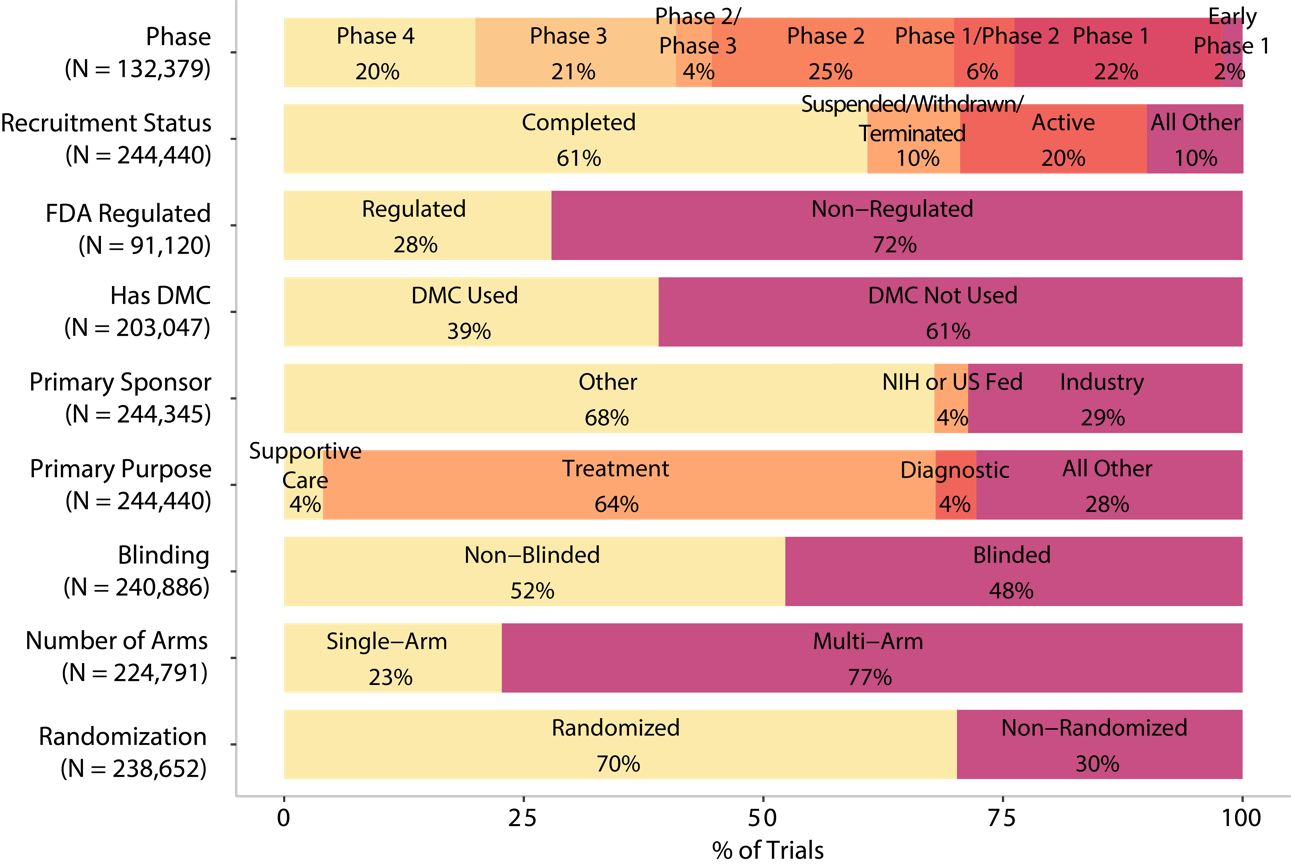
**Supporting Figure S1. **Characteristic of interventional non-oncology trials and associated with COA use. A.** Baseline characteristics of interventional oncology trials. N indicates number of trials using a COA in each category. **B.** Proportion of COA use across trial characteristics.

**A.**

**
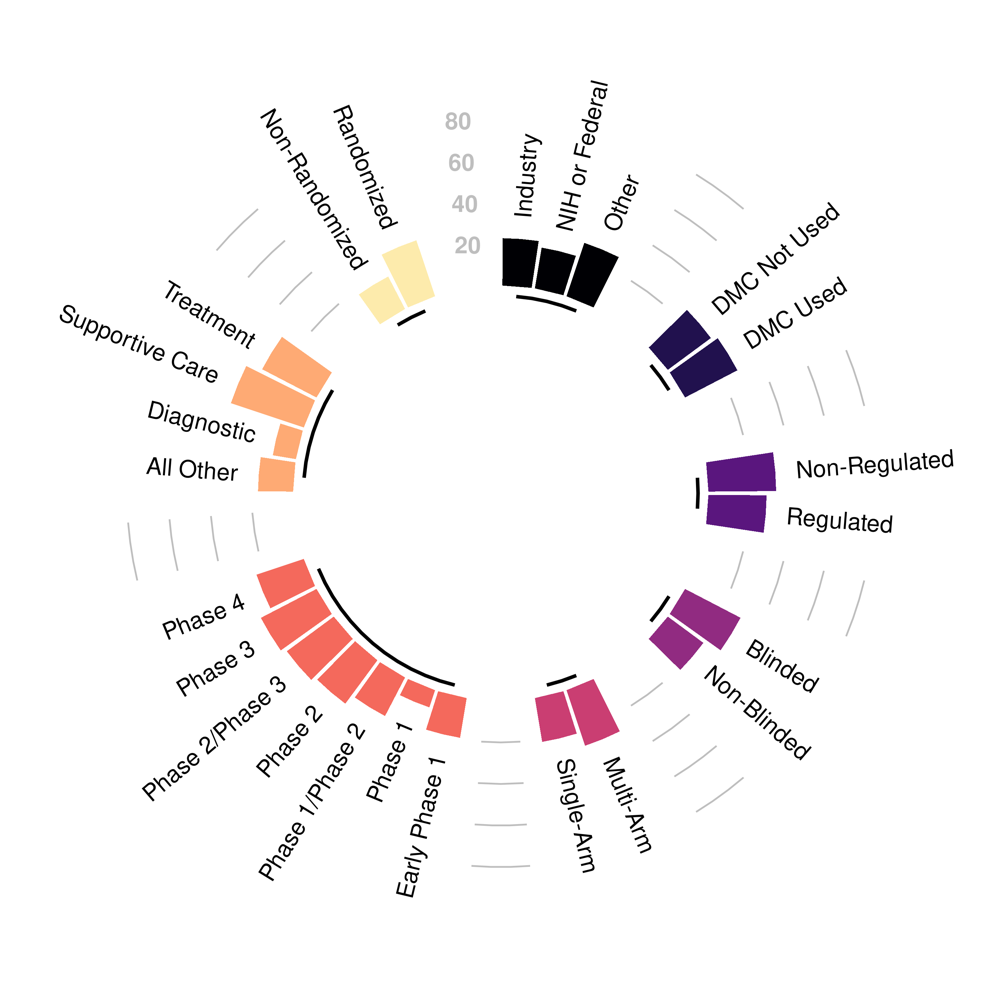
**

**B.**

Supporting Figure S2. **Characteristic of COAs used by interventional non-oncology trials.** **A**. Use in outcomes. **B**. Categories use. **C**. Top COAs used by trials, with COA type and indication.


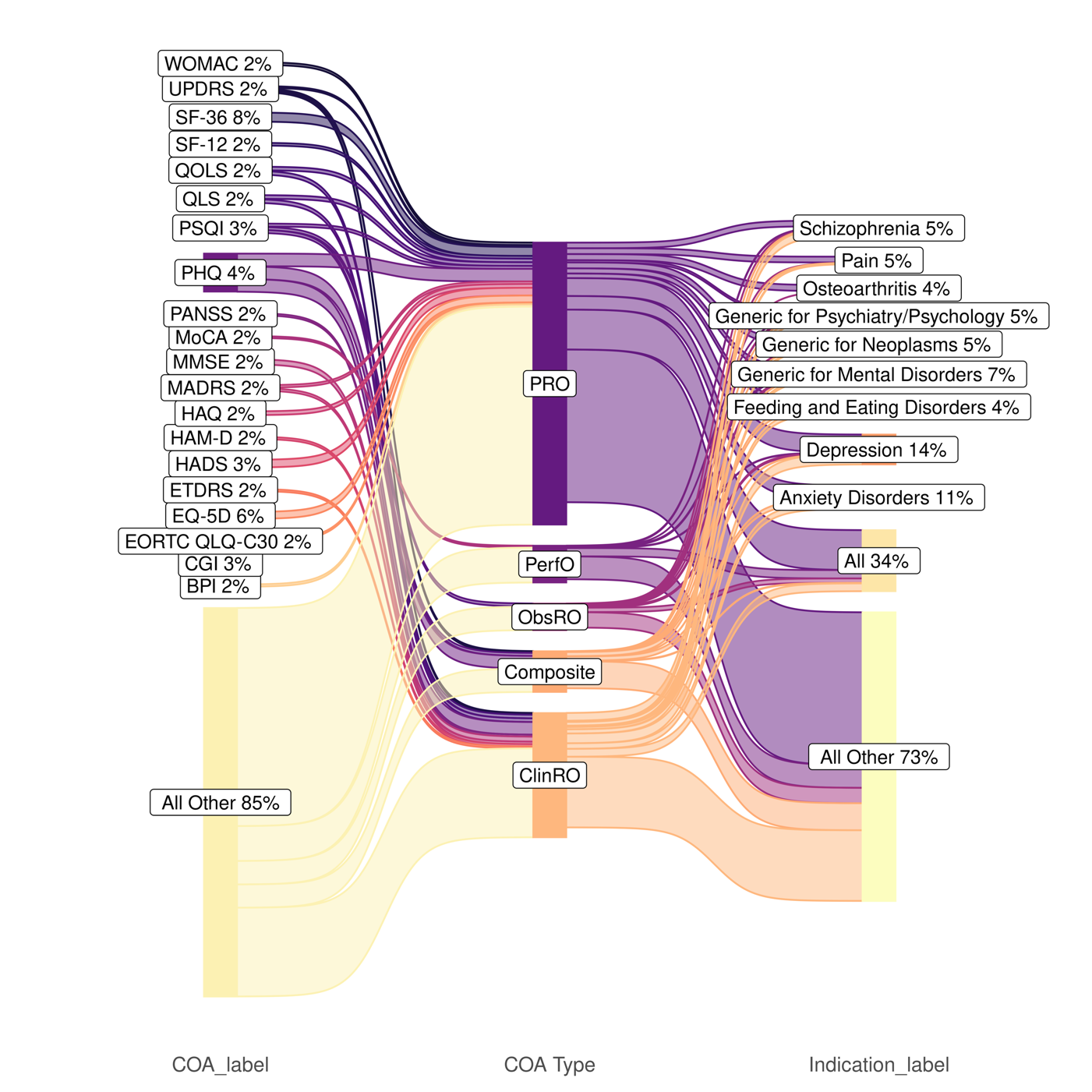


**A.**

**B.**

**C.**


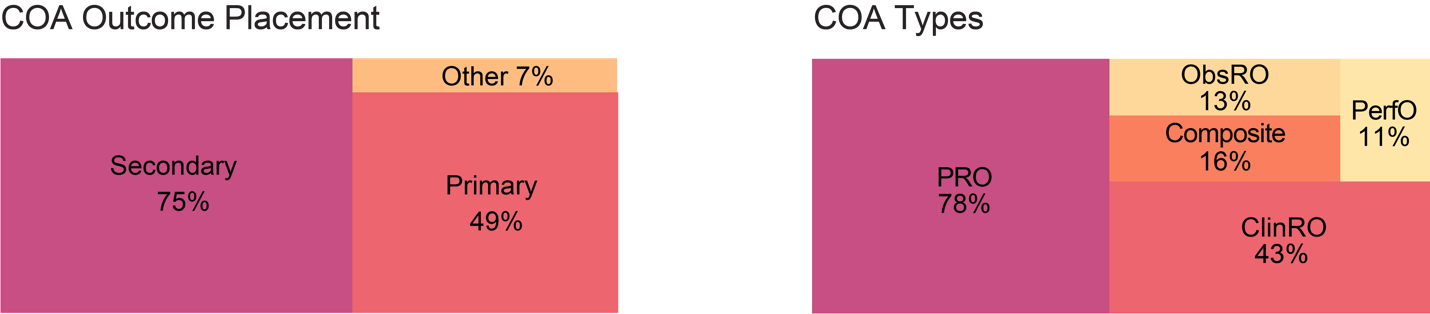


Supporting Figure S3. **Trends of COA use over time among interventional non-oncology trials.** **A**. Linear trend in the number and proportion of trials using COAs across the timeline of notable events for COAs in clinical trials. **B.** Trends in use of Top COAs over time.

**A.**

**B.**


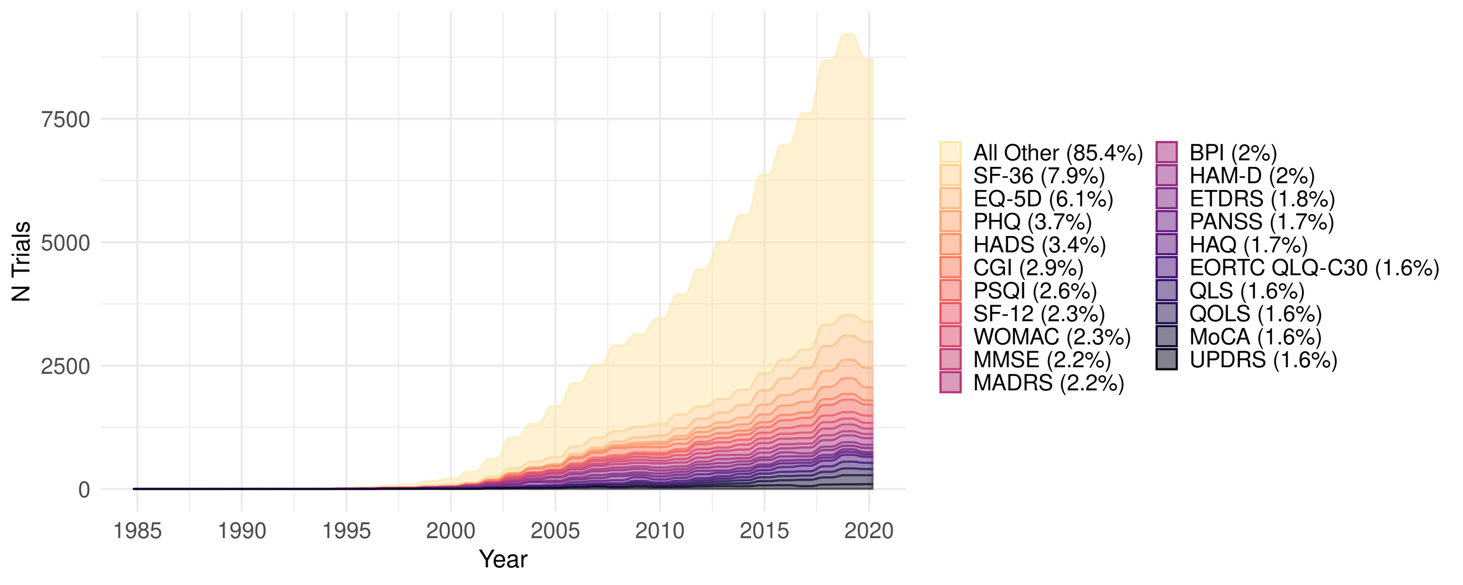

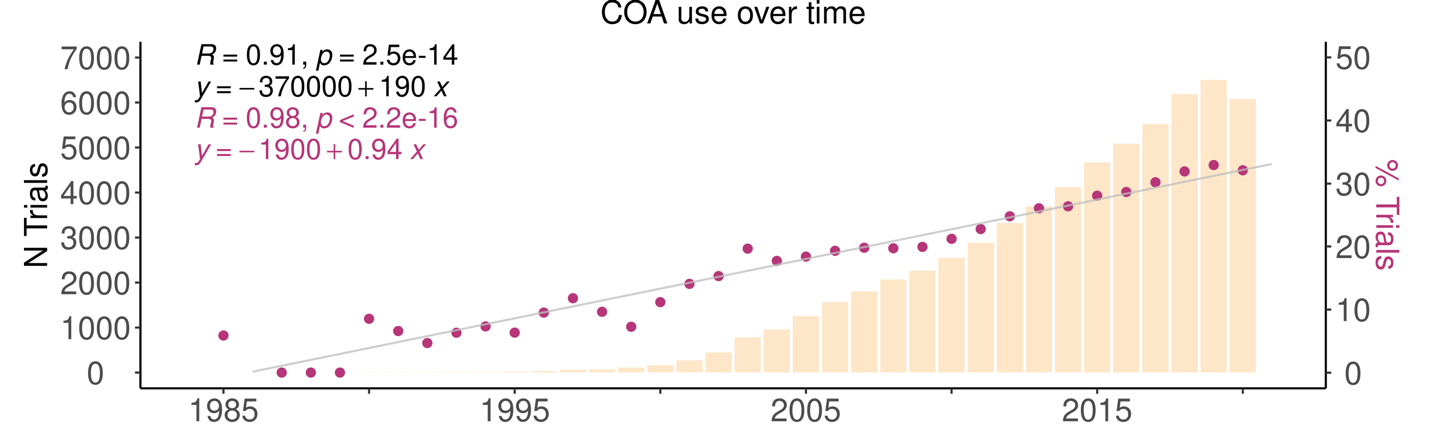


Supporting Figure S4. **Linear trends in the number and proportion of COA use over time stratified by trial primary purpose and COA type.** **A**. Trends among oncology trials. **B.** Trends among non-oncology trials.


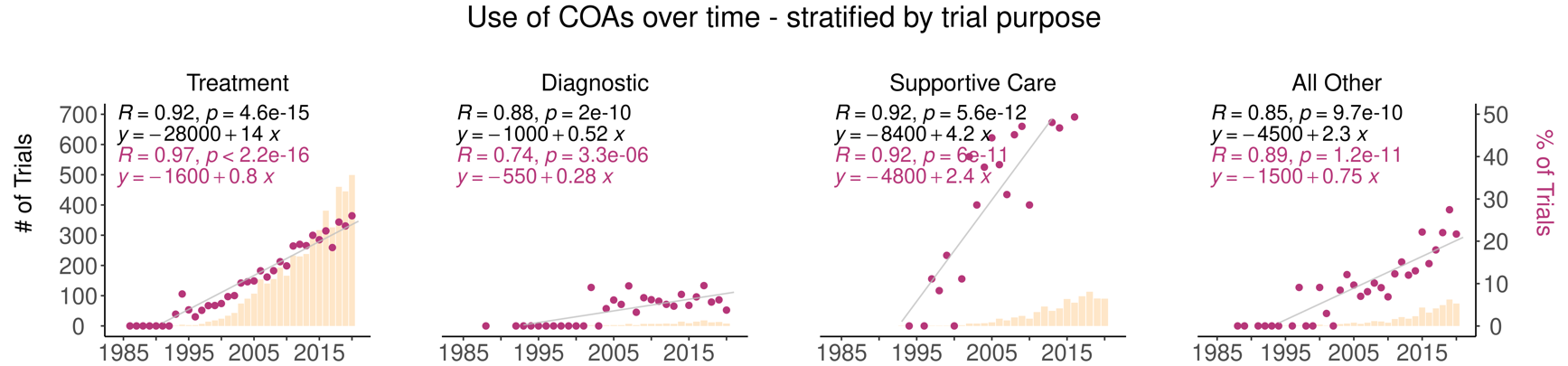
**
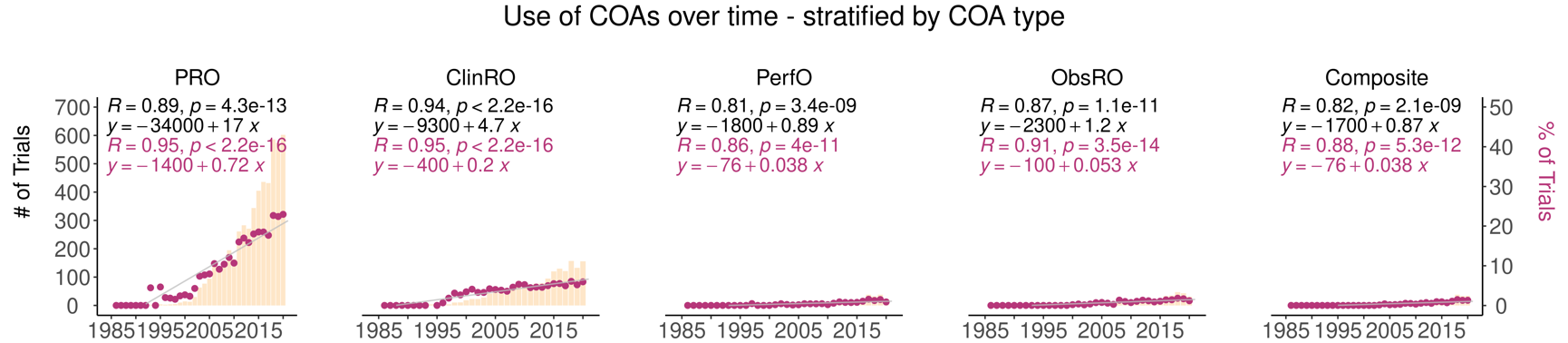
A.**


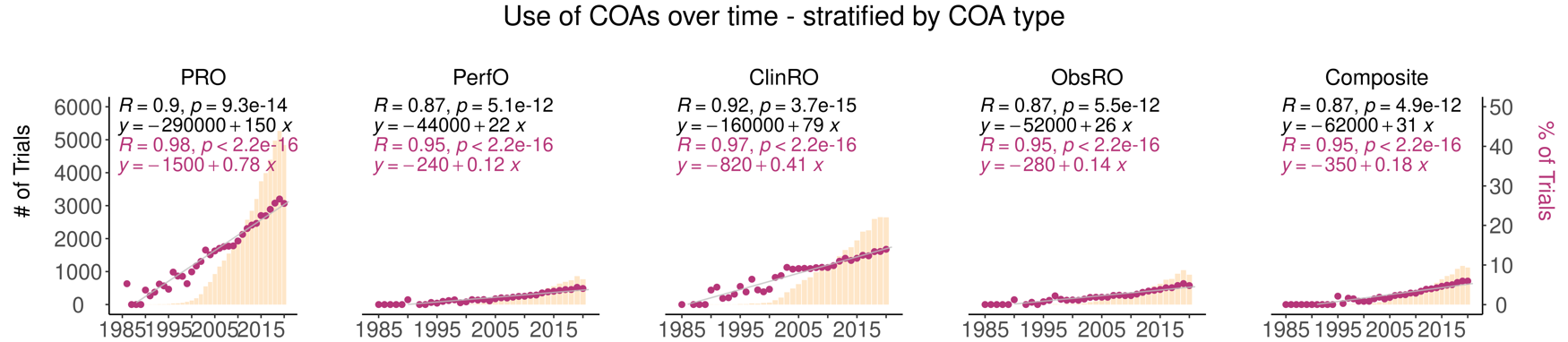

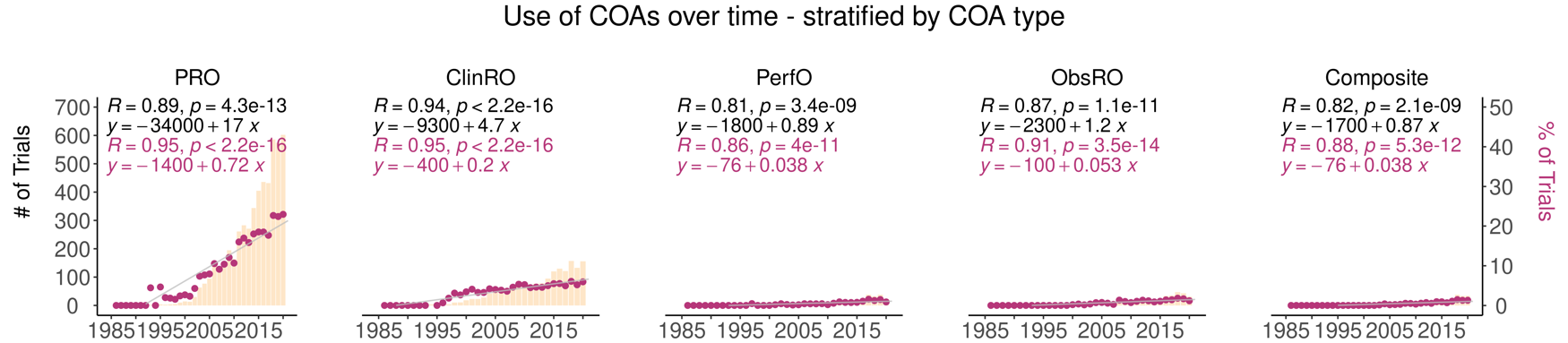
**B**.

Supporting Figure S5. **COA use over time across relevant events.** Grey boxes indicate before versus after event year. **A**. Trends among oncology trials. **B.** Trends among non-oncology trials.


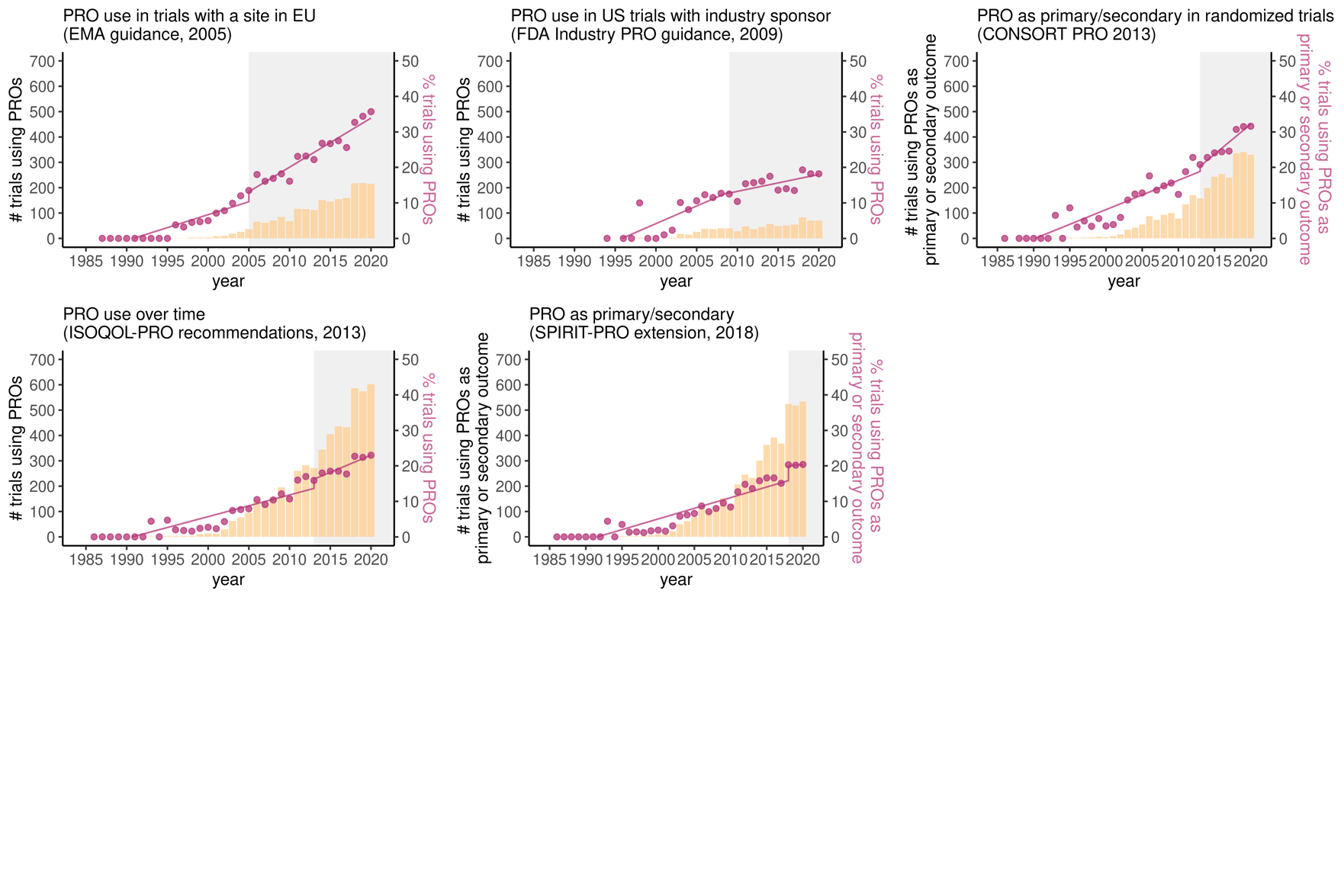
**A.**

**B.**
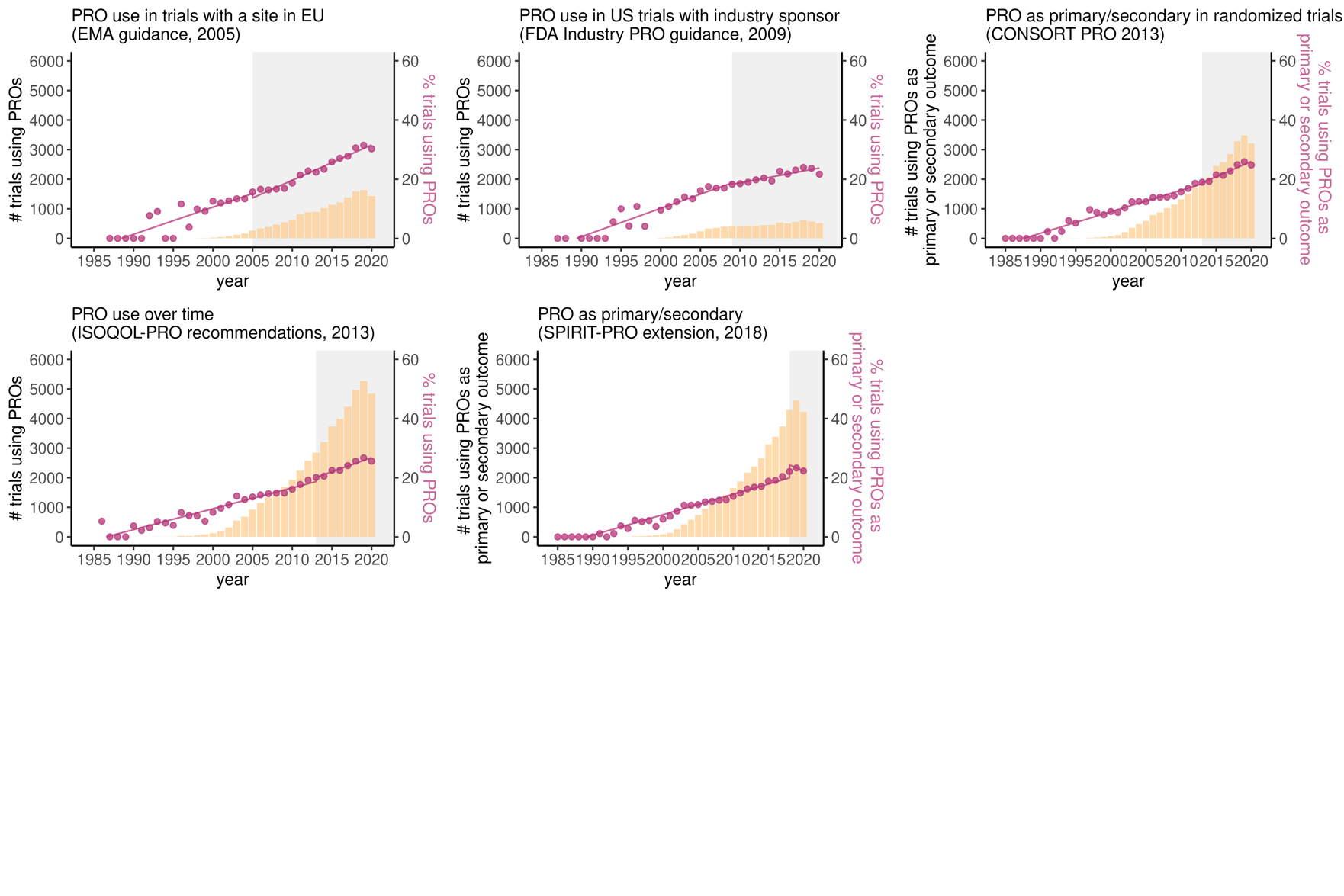

Supplement: Supplementary file 1 — Data S1. Supporting Information [file CAM4-12-16945-s001.docx]
